# Conditional safety margins for less conservative peak local SAR assessment: A probabilistic approach

**DOI:** 10.1002/mrm.28335

**Published:** 2020-06-03

**Authors:** Ettore Flavio Meliadò, Alessandro Sbrizzi, Cornelis A. T. van den Berg, Bart R. Steensma, Peter R. Luijten, Alexander J. E. Raaijmakers

**Affiliations:** ^1^ Department of Radiology University Medical Center Utrecht Utrecht The Netherlands; ^2^ Computational Imaging Group for MR Diagnostics & Therapy, Center for Image Sciences University Medical Center Utrecht Utrecht The Netherlands; ^3^ Tesla Dynamic Coils Zaltbommel The Netherlands; ^4^ Department of Radiotherapy, Division of Imaging & Oncology University Medical Center Utrecht Utrecht The Netherlands; ^5^ Biomedical Image Analysis, Department Biomedical Engineering Eindhoven University of Technology Eindhoven The Netherlands

**Keywords:** parallel transmit, safety factor, SAR model library, SAR model selection, specific absorption rate, subject‐specific local SAR assessment

## Abstract

**Purpose:**

The introduction of a linear safety factor to address peak local specific absorption rate (pSAR_10g_) uncertainties (eg, intersubject variation, modeling inaccuracies) bears one considerable drawback: It often results in over‐conservative scanning constraints. We present a more efficient approach to define a variable safety margin based on the conditional probability density function of the effectively obtained pSAR_10g_ value, given the estimated pSAR_10g_ value.

**Methods:**

The conditional probability density function can be estimated from previously simulated data. A representative set of true and estimated pSAR_10g_ samples was generated by means of our database of 23 subject‐specific models with an 8‐fractionated dipole array for prostate imaging at 7 T. The conditional probability density function was calculated for each possible estimated pSAR_10g_ value and used to determine the corresponding safety margin with an arbitrary low probability of underestimation. This approach was applied to five state‐of‐the‐art local SAR estimation methods, namely: (1) using just the generic body model “Duke”; (2) using our model library to assess the maximum pSAR_10g_ value over all models; (3) using the most representative “local SAR model”; (4) using the five most representative local SAR models; and (5) using a recently developed deep learning–based method.

**Results:**

Compared with the more conventional safety factor, the conditional safety‐margin approach results in lower (up to 30%) mean overestimation for all investigated local SAR estimation methods.

**Conclusion:**

The proposed probabilistic approach for pSAR_10g_ correction allows more accurate local SAR assessment with much lower overestimation, while a predefined level of underestimation is accepted (eg, 0.1%).

## 1 INTRODUCTION

Ultrahigh‐field MRI (UHF‐MRI) provides strong potential to achieve superior image quality compared with the current clinical systems at lower field strengths.^1^, ^2^, ^3^ To address problems with B_1_ homogeneity, often local multitransmit coil arrays are used.^4^, ^5^, ^6^, ^7^, ^8^ However, their application is restricted by the limits of the current methods for local specific absorption rate (SAR) assessment.^9^, ^10^

Multitransmit arrays produce a great variability of the electric field, and thereby of the absorbed power by the tissues,^11^, ^12^, ^13^, ^14^, ^15^, ^16^, ^17^ making the local SAR difficult to predict, which contributes to making the local SAR limits more restrictive than the global SAR limits (as described in IEC 60601‐2‐33).^18^

The local SAR cannot be measured during an MRI examination and is usually evaluated by numerical simulations.^9^, ^10^, ^11^, ^12^, ^13^, ^14^, ^15^, ^16^, ^17^ New software tools to perform on‐line simulations using patient‐specific body models are being developed but are still quite time‐consuming.^19^ Therefore, typically the electric‐field distribution of each array element is simulated off‐line on one or several generic models. After domain reduction by virtual observation points,^20^, ^21^, ^22^ the peak 10*g* average SAR (pSAR_10g_) for a given drive setting of the array is calculated on‐line at the scanner.

Despite the previously mentioned progress, the resulting predicted SAR can still deviate from the true peak local SAR value in the patient being scanned. This is due to a variety of uncertainties in the actual examination setup. Indeed, even assuming that the reflected/lost power is properly monitored, and that there are no calibration errors, the used body model and its position within the MRI system could be very different compared with the patient under examination. To address this, a library of models can be used to cover a large patient population.^10^, ^11^, ^12^, ^17^, ^23^, ^24^, ^25^, ^26^ Nevertheless, the residual uncertainties may still result in peak local SAR overestimation or underestimation error.

Although the overestimation error results in unnecessarily long scan times and/or suboptimal image quality, only peak local SAR underestimation error poses a safety risk. For this reason, to diminish the probability of underestimation, a linear safety factor is usually applied to increase the estimated peak local SAR value to such an extent that underestimation will not occur.^10^, ^12^, ^23^, ^24^ This increased estimated peak local SAR level will be referred to as the “corrected” peak local SAR value.

Recently, an alternative deep learning–based method for subject‐specific SAR estimation was presented.^27^ This data‐driven approach consists of training a convolutional neural network to map the relation between subject‐specific complex $B_{1}^{+}$ maps and the corresponding local SAR distribution. However, like the aforementioned methods, this method also suffers from residual underestimation errors that need to be addressed by applying a suitable safety factor.

As this study will show, the conventionally applied linear safety factor often results in unnecessary very conservative estimation, in particular when high peak local SAR value is estimated. Therefore, we propose an alternative approach to diminish the probability of peak local SAR underestimation based on the conditional probability density function of the true peak local SAR value, given the estimated peak local SAR value. This approach allows us to define a variable safety margin for each possible estimated peak local SAR value.

This approach is applied to five state‐of‐the‐art local SAR estimation methods: local SAR prediction based on (1) one generic model,^25^ (2) the largest value in a large database of models,^11^, ^12^, ^17^ (3) the model that fits best the subject in the scanner,^28^ (4) a combination of methods 2 and 3, and (5) a deep learning method.^27^

In the present work, these methods are applied to assess the peak local SAR with a multitransmit array of eight fractionated dipole antennas for prostate imaging at 7 T.^29^, ^30^ For each method, the mean peak local SAR overestimation is evaluated for the conventional approach using one linear safety factor and the proposed approach, based on the probability density function. The results show that the proposed approach is better able to deal with the remaining uncertainty and reduces peak local SAR overestimation with respect to all other investigated local SAR estimation methods. In addition to the introduction of the conditional safety margin (CSM) approach based on the probability density function, this study presents a comparative evaluation of five potential local SAR assessment techniques that were described previously.

## 2 THEORY

**Figure 1:**
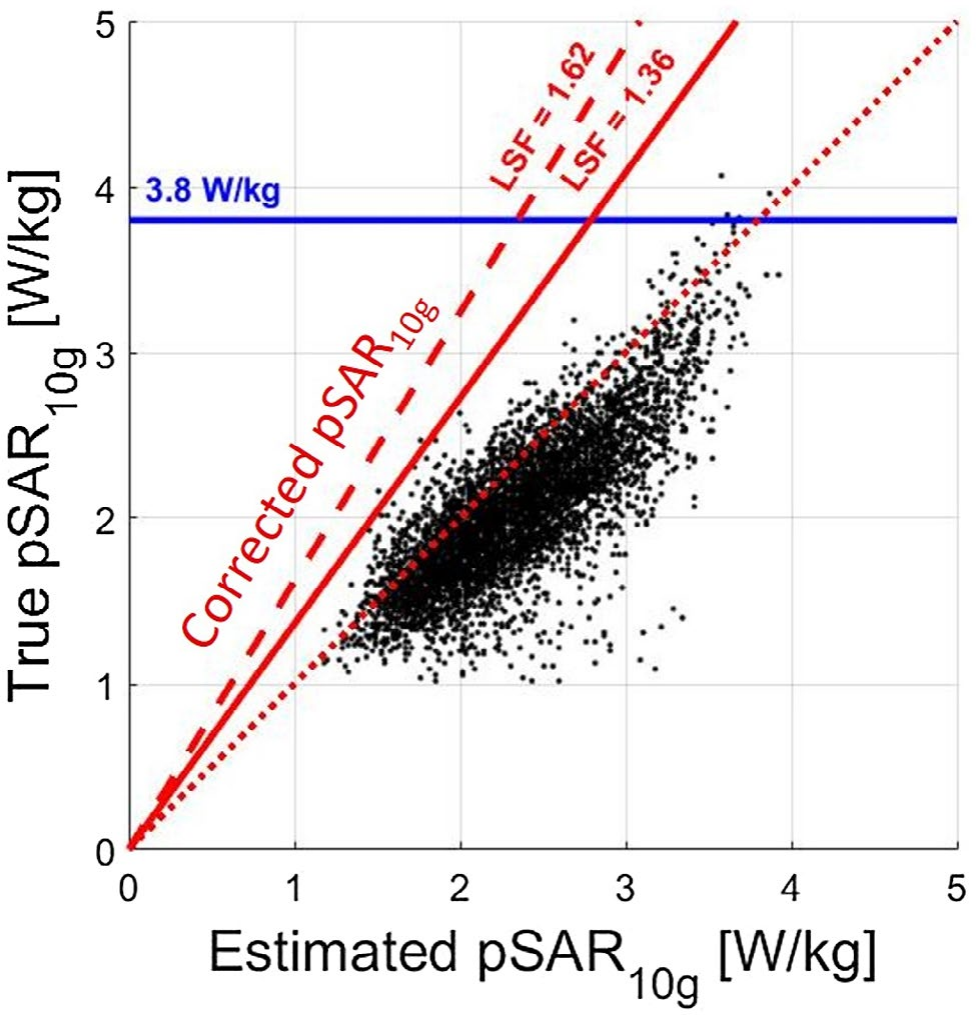
Scatter plot of the true peak local specific absorption rate (pSAR_10g_) versus the estimated pSAR_10g_ for 7T prostate imaging using a wide variety of random phase settings and 23 human models. The estimated pSAR_10g_ is the pSAR_10g_ value as determined by the deep learning–based method^27^ using simulated $B_{1}^{+}$ distributions as input. The true pSAR_10g_ is given by the simulated pSAR_10g_ value. The diagonal red dotted line indicates where the estimated pSAR_10g_ and true pSAR_10g_ values are equal. The dashed red line denotes the corrected pSAR_10g_ using the linear safety factor (LSF), defined to avoid underestimation errors in all cases evaluated (LSF = 1.62). The solid red line is the corrected pSAR_10g_ by the LSF defined by removing the outliers (LSF = 1.36). The blue line is the 99.9% certain pSAR_10g_ upper bound (3.8 W/kg) for random phase settings

The introduction of a linear safety factor to avoid peak local SAR (pSAR_10g_) underestimation bears one considerable drawback: If the estimated pSAR_10g_ level is high, then the resulting corrected pSAR_10g_ level shows severe overestimation of the true pSAR_10g_ level. This is depicted in Figure 1. This figure shows for a certain transmit array configuration how estimated pSAR_10g_ (pSAR^E^) and truly obtained pSAR_10g_ (pSAR^T^) are related. In particular, each point in the scatter plot represents one out of 5750 pSAR_10g_ estimations by the deep learning method with 8 × 1W input power and random phase shimming. The diagonal red dotted line indicates where the estimated pSAR_10g_ levels and true pSAR_10g_ levels are equal. Points above the diagonal represent true pSAR_10g_ levels larger than the corresponding estimate. The scatter plot clearly shows that there is a good correlation between estimated pSAR_10g_ level and true pSAR_10g_ level. However, the estimated pSAR_10g_ level needs to be corrected to avoid underestimation, as there are many points above the dotted line. A linear safety factor of 1.62 is therefore determined to correct (increase) the estimated pSAR_10g_ level, such that all of the potential true pSAR_10g_ levels are lower than the corrected pSAR_10g_ level.

The red dashed line shows how each estimated pSAR_10g_ level on the horizontal axis results in a corrected pSAR_10g_ (pSAR^E,C^) level. If the correction factor is chosen appropriately, all points will be below the dashed red line. As Figure 1 shows, this condition is fulfilled.

However, the figure also clearly illustrates that for large estimated pSAR_10g_ levels, the application of the linear safety factor results in corrected pSAR_10g_ levels that have far too high overestimation. The overestimation could be reduced by determining the safety factor using the upper outer fence.^27^ This will make the safety factor less sensitive to outliers and/or small deviations in estimated or true pSAR_10g_ of the one point that determines its slope. Then, using the upper outer fence definition,^27^ the linear safety factor will be 1.36, as indicated by the solid red line in Figure 1. The figure shows that more than 99.9% of the points are below the line.

Unfortunately, for large estimated pSAR_10g_ levels, the corrected pSAR_10g_ level is still much larger than what realistically could be expected. In fact, a previous study^12^ has shown that the pSAR_10g_ level that is not exceeded for 99.9% of the cases (over all 23 models and any potential phase setting) is 3.8 W/kg. Therefore, a solution could be to combine these limits that use the corrected pSAR_10g_ value if the value is lower than 3.8 W/kg, and set it equal to 3.8 W/kg otherwise (blue line in Figure 1).

**Figure 2:**
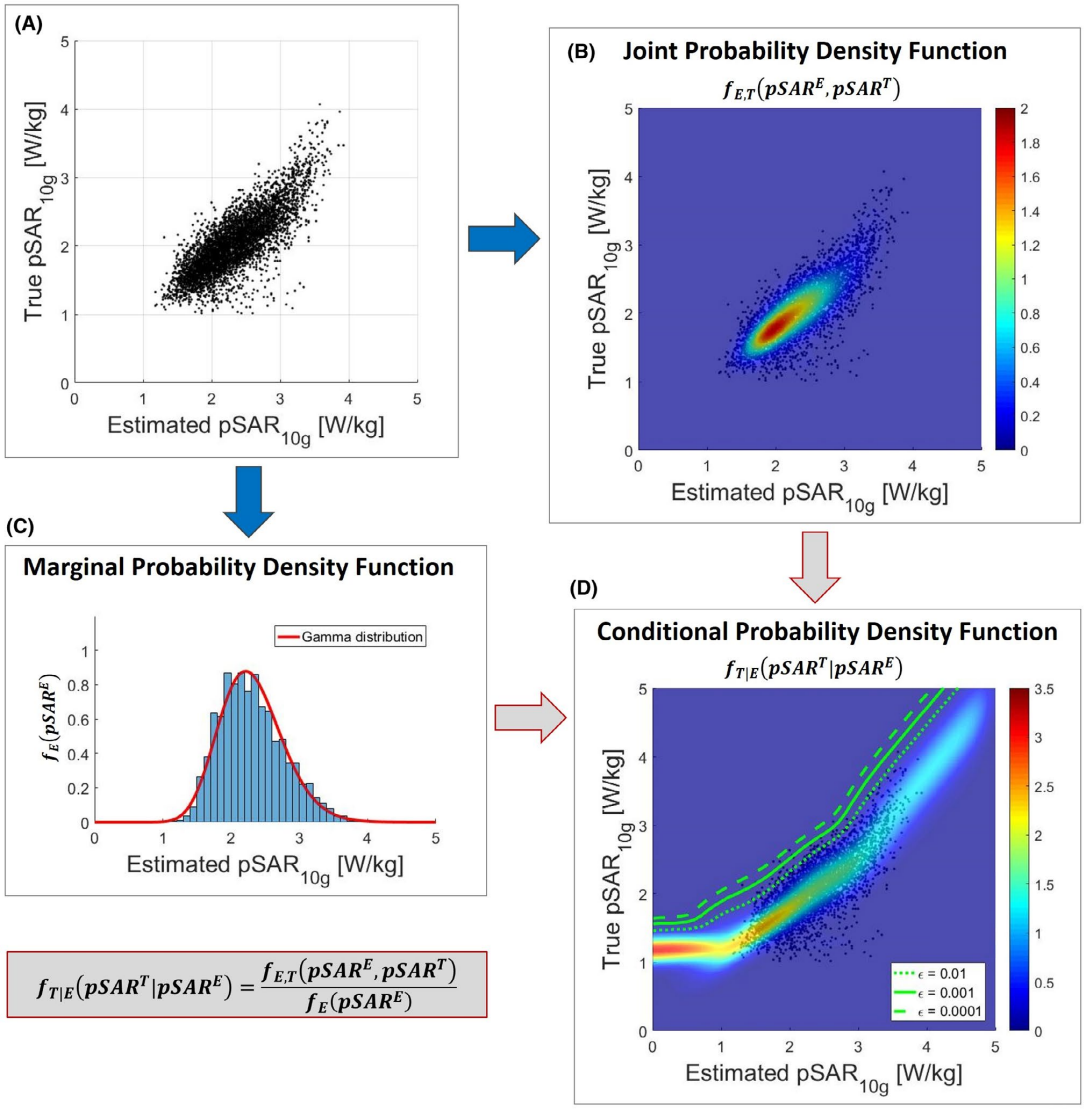
A, Scatter plot of the true pSAR^T^ versus the estimated pSAR^E^ using the deep learning method. B, Joint probability density function *f_E,T_(*pSAR^E^, pSAR^T^ *)* of the estimated and true pSAR values. C, Marginal probability density function *f_E_(*pSAR^E^ *)* of the estimated pSAR values. D, Two‐dimensional conditional probability density function *f_T|E_(*pSAR^T^ *|*pSAR^E^ *)* obtained by combining the conditional probability density functions for each possible pSAR^E^ value

However, from a mathematical perspective, this approach is arguably not the most appropriate solution to address this problem. Instead, a probabilistic approach should be followed. In a probabilistic setting, the scatter plot in Figure 1 represents samples from the joint probability distribution of estimated and true pSAR_10g_ values, which is described by the probability density function *f_E,T_* (*pSAR^E^*, *pSAR^T^*). When a pSAR^E^ value is estimated, we would like to know the probability of pSAR_10g_ underestimation: *P*(*pSAR^T^*^^ *>* *pSAR^E^*). Thus, one needs to know the conditional probability density function *f_T|E_* (*pSAR^T^|pSAR^E^*), which describes the probability distribution of pSAR^T^ for a given pSAR^E^ value. This function is calculated in Eq. 1, where *f_E_* (*pSAR^E^*) is the marginal probability density function that describes the probability distribution of estimating the *pSAR^E^* value regardless of *pSAR^T^* (Figure 2).

(1) $$f_{T|E}\left(pSAR^{T}|pSAR^{E}\right)=\frac{f_{E,T}\left(pSAR^{E},\;pSAR^{T}\right)}{f_{E}\left(pSAR^{E}\right)}$$

Now the probability of pSAR_10g_ underestimation (*P_underest_*) (ie, the probability that the true value pSAR^T^ is actually larger than some estimated value *pSAR^E^* *=* *E*_1_) is given as

(2) $$P_{\mathrm{underest}}=P\left(pSAR^{T}>E_{1}|pSAR^{E}=E_{1}\right)=\int_{E_{1}}^{\infty }f_{T|E}\left(pSAR^{T}|pSAR^{E}=E_{1}\right)d\left(pSAR^{T}\right).$$

Therefore, the conditional probability density function can be calculated for the estimated *pSAR^E^* = *E*_1_ value, and the integral can be numerically evaluated to determinate a corrected *pSAR^E,C^* value with probability of underestimation equal to an arbitrary (small) *ε*:

$FindpSAR^{E,C}such\;\;that:$

(3) $$P\left(pSAR^{T}>pSAR^{E,C}|pSAR^{E}=E_{1}\right)=\int_{pSAR^{E,C}}^{\infty }f_{T|E}\left(pSAR^{T}|pSAR^{E}=E_{1}\right)d\left(pSAR^{T}\right)=\varepsilon .$$

This corrected pSAR^E,C^ value effectively results in a CSM that depends on the estimated pSAR_10g_ level and that can be determined for each possible estimated *pSAR^E^* value.

Note that, from the observed data, a variety of approaches can be used to estimate the underlying probability density function,^31^ including kernel density estimator,^32^ histogram,^33^ and mixture models.^34^

## 3 METHODS

The exact extent of reduction of overestimation using the proposed approach depends on the method of pSAR_10g_ estimation. In this study, the pSAR_10g_ values were predicted using five state‐of‐the‐art local SAR estimation methods. Then, for each estimation method, the corrected pSAR_10g_ values were obtained by applying the linear safety factor and the proposed approach based on probability density function.

To build the required set of (pSAR^T^, pSAR^E^) samples for the purpose of this study, our database of 23 subject‐specific models^12^ with an 8‐channel transmit body array for 7T prostate imaging^29^, ^30^ was used. For each model and every array channel, finite‐difference time‐domain simulations were performed (Sim4Life; ZMT, Zürich, Switzerland), and the results were processed to obtain the true pSAR_10g_ and the estimated pSAR_10g_ by each method, when the transmit array is driven with different drive vectors.

Three driving modes were considered:

- Random phase settings: Drive vectors with uniform amplitude (8 × 1W input power) and random relative phase settings with respect to the first channel (uniform distribution: $U\left(-180^{\circ },180^{\circ }\right)$). This driving mode allows one to assess the performances during an arbitrary imaging examination in the lower abdomen region.
- Prostate shimmed phase settings: Drive vectors with uniform amplitude (8 × 1W input power) and relative phase normally distributed around the average prostate phase shimming set^12^ $N\left(\mu =0,\sigma =33^{\circ }\right)$. This driving mode allows one to assess the likely performances during a prostate imaging examination.
- Random amplitudes and phases settings: Drive vectors with random amplitude ( $U\left(0,1\right)$) and random relative phase settings ( $U\left(-180^{\circ },180^{\circ }\right)$). This driving mode allows one to assess the performances even in the case of sophisticated RF pulse design strategies (eg, SPINS.^5^ *k*_T_‐points^6^). Two scenarios were investigated: one in which the drive vectors were normalized to 1W maximum input power per channel, and one in which the drive vectors were normalized to 8W total input power.

### 3.1 Peak local SAR correction

#### 3.1.1 Linear safety factor

For each model, 250 different drive vectors were used to calculate the true and the estimated pSAR_10g_ values. Then, for each pSAR_10g_ estimation method, 5750 (23 × 250) validation sets are used to determine the required linear safety factor using the upper outer fence method^27^ as described in the section 2.

The safety factor application can produce very highly corrected pSAR_10g_ values, particularly for high estimated pSAR_10g_ levels. In our previous study,^12^ based on the probability distribution of the true pSAR_10g_, we defined a 99.9% certain pSAR_10g_ upper bounds of 3.8 W/kg and 3.2 W/kg for random phases and prostate shimmed phases, respectively. In the same way, we can define a 99.9% certain pSAR_10g_ upper bound of 2.6 W/kg and 6.8 W/kg for random amplitudes and phase settings normalized to 1W maximum input power per channel and 8W total input power, respectively. Therefore, as explained in section 2, we can further reduce overestimation of the linear safety factor approach by combining it with these pSAR_10g_ upper bounds, when higher corrected pSAR_10g_ values are obtained (Figure 1, blue line).

#### 3.1.2 Conditional safety margin

We assumed that the 23 models are representative of the entire patient population. Then, for each pSAR_10g_ estimation method, the pairs‐estimated and true pSAR_10g_ values (*pSAR^E^*, *pSAR^T^*) are samples from the joint probability density function *f_E,T_* (*pSAR^E^*, *pSAR^T^*) (Figure 2A) that can be modeled as a Gaussian mixture (Figure 2B). The estimated pSAR_10g_ values are samples from the marginal probability density function *f_E_* (*pSAR^E^*) and follow what appears to be a gamma distribution (Figure 2C).

Therefore, the conditional probability density function can be calculated, and the CSM with an arbitrary small probability of underestimation ε ( ε=0.001in our study) can be determined using Equation 3 (Figure 2D).

For the practical implementation of the proposed approach, the domain *f_E,T_* (*pSAR^E^*, *pSAR^T^*) is discretized (eg, ∆(*pSAR^E^*), ∆ (*pSAR^T^*) = 0.01 W/kg) and the estimated statistical models are used to obtain the 2D array of *f_E,T_* (*pSAR^E^*, *pSAR^T^*) values and the 1‐dimensional array of *f_E,T_* (*pSAR^E^*) values. Then, from the ratio between each column of the first array and the corresponding value of the second array, the 2D array of *f_,T|E_* (*pSAR^T^*|*pSAR^E^*) values are determined. Each column of the obtained array represents the conditional probability density function for the corresponding *pSAR^E^* value, and the CSM is determined by integrating numerically along the column until the required probability of underestimation is reached (the integral along the entire column will be equal to 1). Finally, the obtained CSMs for the discretized *pSAR^E^* values are interpolated on‐line to determine the CSM for any possible estimated *pSAR^E^* value.

### 3.2 True peak local SAR

To obtain the true pSAR_10g_ value, for each model *m* and each drive setting ***s***, the 10*g*‐averaged *Q*‐matrices (***Q***_10 g_)^20^, ^21^ are calculated as follows:

(4) $$pSAR_{m,s}^{T}=\max_{r}\left(s^{H}Q_{10g}{\left(r\right)}_{m}s\right),$$

where r is the spatial location of each ***Q***_10 g_ matrix.

### 3.3 Peak local SAR estimation methods

For each model *m* and each drive setting ***s***, the corresponding pSAR_10g_ values are estimated using five different methods.

#### 3.3.1 Generic body model

A very common approach consists of performing off‐line electromagnetic simulations using a generic body model. Then, assuming that the investigated model is representative for the current subject, on‐line pSAR_10g_ estimation based on the actual drive scheme can be performed.

To assess the performance of this method, the generic model “Duke” of the Virtual Family^25^ with 77 tissues (version 3.0; voxel resolution 0.5 × 0.5 × 0.5 mm^3^) is used as follows:

(5) $$pSAR_{m,s}^{E,GM}=\max_{r}\left(s^{H}Q_{10g}{\left(r\right)}_{\mathrm{Duke}}s\right).$$

#### 3.3.2 Model library

To cover an entire population of patients, a model library can be used to predict the maximum pSAR_10g_ over all models.^11^, ^12^, ^17^ Therefore, for each model *m*, and each drive setting ***s***, the maximum pSAR_10g_ over the other models is determined as reported in Equation 6:

(6) $$pSAR_{m,s}^{E,ML}=\max_{k\neq m}\left(pSAR_{k,s}^{T}\right).$$

#### 3.3.3 Model selection

The estimation error might be reduced using a large database of models and selecting the most representative local SAR model for the patient under examination.^17^ However, in our previous work,^12^ no relationship was found between measurable body features and pSAR_10g_. Therefore, as presented in our preliminary study,^28^ for each model *m* we consider the model *n*, which shows the most similar worst‐case local SAR distribution with uniform amplitude ${\mathrm{SAR}}_{10g}^{\mathrm{WoC}}$ (ie, for each voxel, the maximum SAR level that could possibly be achieved with 8 × 1W input power and worst‐case phase settings),^35^ as its most representative local SAR model. Indeed, the ${\mathrm{SAR}}_{10g}^{\mathrm{WoC}}$ distribution contains both patient anatomy information and electric‐field information (transmit‐array information). In particular, we select the model *n*, which presents the ${\mathrm{SAR}}_{10g}^{\mathrm{WoC}}$ distribution that minimized the RMS error with respect to the registered ${\mathrm{SAR}}_{10g}^{\mathrm{WoC}}$ distribution of the model *m* (Supporting Information Figures S1‐S3 report the ${\mathrm{SAR}}_{10g}^{\mathrm{WoC}}$ distributions, the RMS error matrix, and the most representative local SAR models). Then, for each drive setting ***s***, the pSAR_10g_ value is estimated using model *n* as follows:

(7) $$pSAR_{m,s}^{E,MS}=pSAR_{n,s}^{T}.$$

#### 3.3.4 Multiple models selection

As results will show, the model library method results in large mean overestimation, while the model selection method has large underestimation errors. For these reasons, a good compromise could be the selection of multiple models.

Using the same model selection approach of the previous method, for each model *m*, the group of five most representative local SAR models *A_m_* is identified (Supporting Information Table S1), and the maximum pSAR_10g_ of these five models is used to determine the performance of the multiple models selection method as follows:

(8) $$pSAR_{m,s}^{E,MMS}=\max_{k\in A_{m}}\left(pSAR_{k,s}^{T}\right).$$

#### 3.3.5 Deep learning–based method

The deep learning–based method is a new image‐based method.^27^ In this data‐driven approach, a convolutional neural network is trained to learn a “surrogate SAR model” to map the relation between subject‐specific complex $B_{1}^{+}$ maps and the corresponding local SAR distribution.

Therefore, for each model *m* and each drive setting ***s***, the simulated $B_{1}^{+}$ maps of each channel *i* are processed to produce the shimmed $B_{1}^{+}$ map as follows:

(9) $$B_{1}^{+}{\left(r\right)}_{m,s}=\sum_{i=1}^{Nch.}B_{1}^{+}{\left(r,\;\;i\right)}_{m}s\left(i\right),$$

where *Nch*. = 8 is the number of transmit channels. Then, with the obtained complex $B_{1}^{+}$ maps, realistic synthetic MR images are generated (magnitude and relative transmit phase maps with noise)^27^ and used to produce the input data for the trained convolutional neural network to infer the corresponding SAR_10g_ distribution and determine the peak value, as follows:

(10) $$SAR_{10g}^{\mathrm{DL}}{\left(r\right)}_{m,s}=CNN\left(B_{1}^{+}{\left(r\right)}_{m,s}\right)$$

(11) $$pSAR_{m,s}^{E,DL}=\max_{r}\left(SAR_{10g}^{\mathrm{DL}}{\left(r\right)}_{m,s}\right)$$

Note that the performance of the deep learning–based method is evaluated by performing a leave‐one‐out cross‐validation. This means that 23 separate times, the convolutional neural network is trained on all of the data samples from all models except for one model (22 × 250 = 5500 training samples with random phase shimming).

### 3.4 Performance evaluation

To assess the performance of all methods covered by this study, for each body model, 1000 test sets were generated for each driving mode. Then, using the obtained 23 000 (23 × 1000) test sets, the mean pSAR_10g_ overestimation error of all pSAR_10g_ estimation methods with the linear safety factor and with the proposed CSM were evaluated and compared with each other for each driving mode.

## 4 RESULTS

For each pSAR_10g_ estimation method and for each driving mode, 5750 (23 × 250) validation sets were used to define the required linear safety factor and the CSM. For random phase and prostate shimmed‐phase drive modes, the required linear safety factors were similar and ranged from 1.36 for the deep learning method to 1.96 for the model selection method. For the amplitude and phase shimming driving mode, the obtained linear safety factors range was a bit wider, ranging from 1.22 to 2.09 for deep learning and model selection methods, respectively.

To determine the joint and marginal probability density functions of each pSAR_10g_ estimation method, each scatter plot of true versus estimated pSAR_10g_ was fitted with a Gaussian mixture distribution (the number of Gaussian terms was determined with the Bayesian information criterion^36^ and ranged between 3 and 5), and the estimated pSAR_10g_ histogram was fitted with a gamma distribution (*MATLAB* and *Statistics* *and* *Machine* *Learning* *Toolbox*, The MathWorks, Natick, MA). Subsequently, the conditional probability density function was determined and the CSM was evaluated to have a probability of underestimation of 0.1% ( ε=0.001). All probability density functions are reported in Supporting Information Figures S4‐S8).

The performances of the defined safety factors were assessed for each pSAR_10g_ estimation method and each driving mode with 23 000 (23 × 1000) test sets.

**Figure 3:**
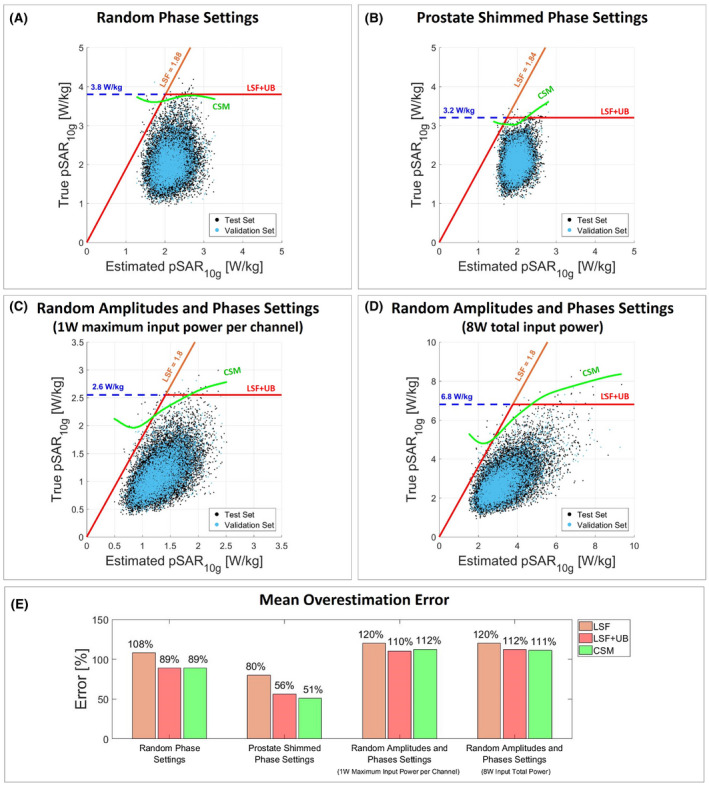
Generic body model. Scatter plot of true versus estimated pSAR_10g_ values with random phases (A), prostate shimmed phase settings (B), random amplitudes and phases normalized to 1W maximum input power per channel (C), and random amplitudes and phases normalized to 8W input total power (D). The orange line denotes the corrected pSAR_10g_ by the LSF; the red line denotes the corrected pSAR_10g_ by the LSF limited by the 99.9% certain pSAR_10g_ upper bound (UB) (dashed blue line); and the green line denotes the corrected pSAR_10g_ by the conditional safety margin (CSM). The LSF and the CSM are determined using the validation set (validation set: cyan dots; vest set: black dots). E, Bar plot of the mean pSAR_10g_ overestimation error for each pSAR_10g_ correction method and each driving mode

Figure 3 shows the scatter plot of true versus estimated pSAR_10g_ considering the use of a single generic body model to evaluate the pSAR_10g_ with random phases (Figure 3A), prostate shimmed phases (Figure 3B) and random amplitudes and phases normalized to 1W maximum input power per channel (Figure 3C), and 8W total input power (Figure 3D). The orange line denotes the corrected pSAR_10g_ after the application of the linear safety factor. The red line denotes the corrected pSAR_10g_ after application of the linear safety factor limited by the 99.9% certain pSAR_10g_ upper bound (dashed blue line). The green line denotes the corrected pSAR_10g_ using the CSM. Figure 3E shows the mean overestimation for each pSAR_10g_ correction method.

**Figure 4:**
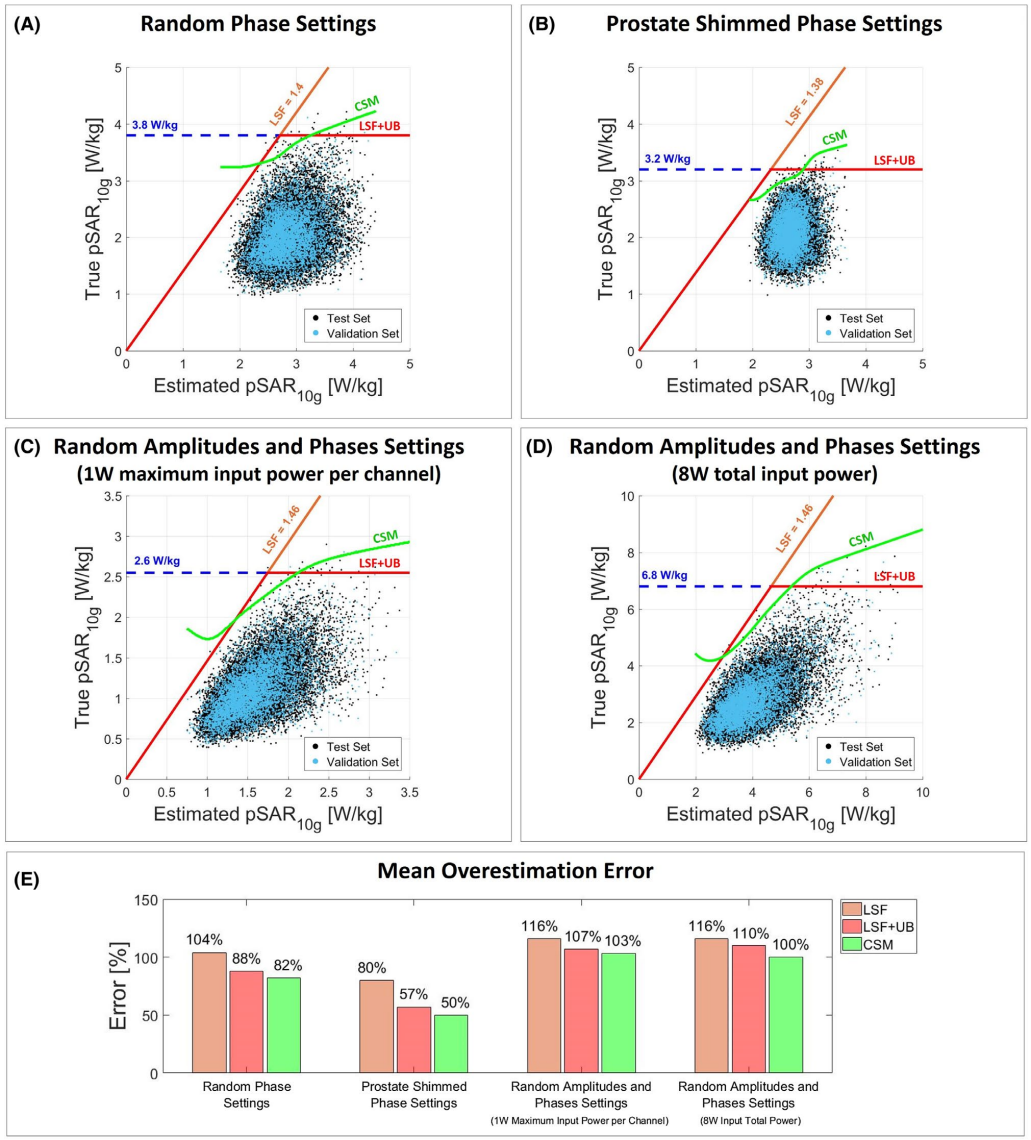
Model library. Scatter plot of true versus estimated pSAR_10g_ with random phases (A), prostate shimmed phase settings (B), random amplitudes and phases normalized to 1W maximum input power per channel (C), and random amplitudes and phases normalized to 8W input total power (D). The orange line denotes the corrected pSAR_10g_ by the LSF; the red line denotes the corrected pSAR_10g_ by the LSF limited by the 99.9% certain pSAR_10g_ upper bound (dashed blue line); and the green line denotes the corrected pSAR_10g_ by the CSM. The LSF and the CSM are determined using the validation set (validation set: cyan dots; test set: black dots). E, Bar plot of the mean pSAR_10g_ overestimation error for each pSAR_10g_ correction method and each driving mode

**Figure 5:**
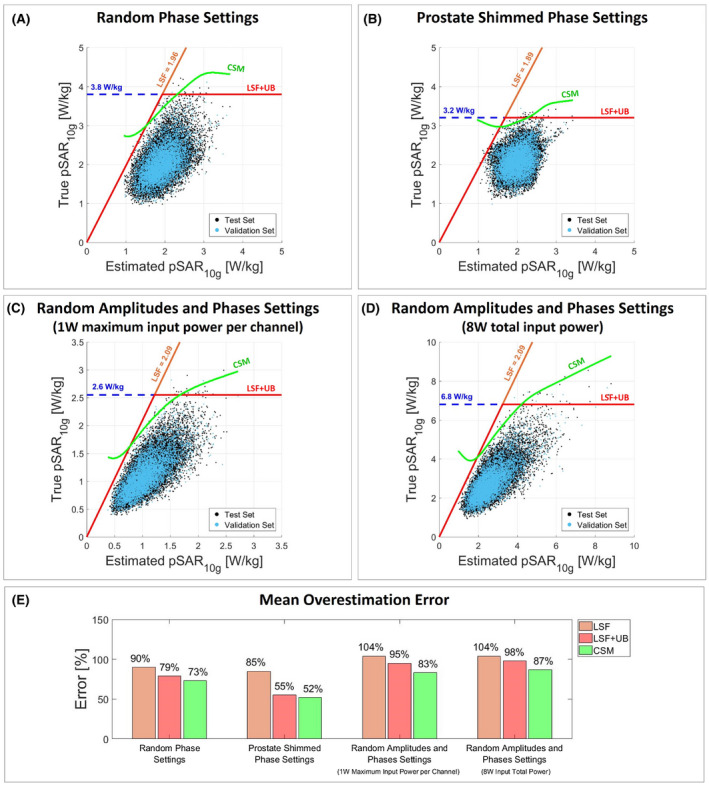
Model selection. Scatter plot of true versus estimated pSAR_10g_ with random phases (A), prostate shimmed phase settings (B), random amplitudes and phases normalized to 1W maximum input power per channel (C), and random amplitudes and phases normalized to 8W input total power (D). The orange line denotes the corrected pSAR_10g_ by the LSF; the red line denotes the corrected pSAR_10g_ by LSF limited by the 99.9% certain pSAR_10g_ upper bound (dashed blue line); and the green line denotes the corrected pSAR_10g_ by the CSM. The LSF and the CSM are determined using the validation set (validation set: cyan dots; test set: black dots). E, Bar plot of the mean pSAR_10g_ overestimation error for each pSAR_10g_ correction method and each driving mode

**Figure 6:**
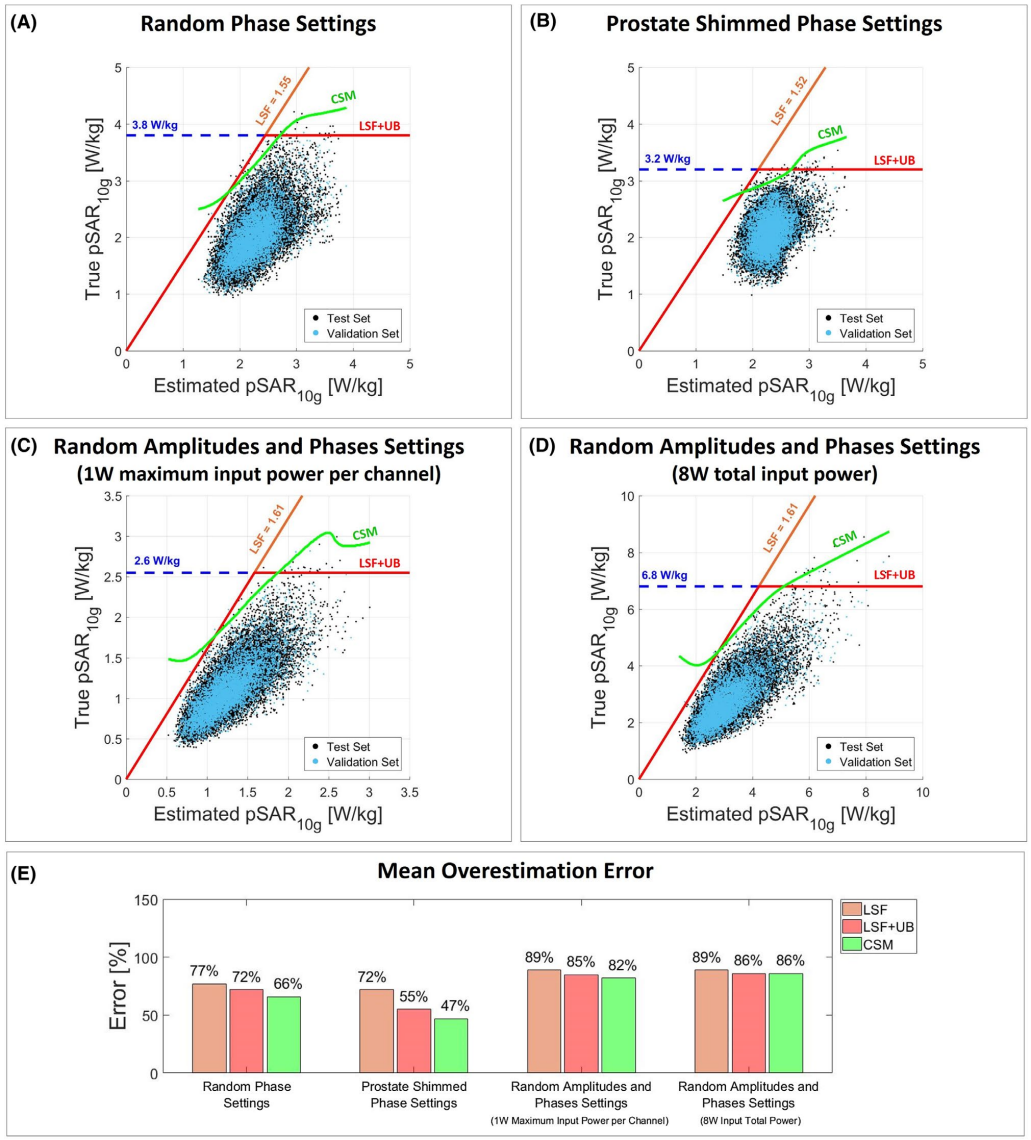
Multiple models selection. Scatter plot of true versus estimated pSAR_10g_ with random phases (A), prostate shimmed phase settings (B), random amplitudes and phases normalized to 1W maximum input power per channel (C), and random amplitudes and phases normalized to 8W input total power (D). The orange line denotes the corrected pSAR_10g_ by the LSF; the red line denotes the corrected pSAR_10g_ by the LSF limited by the 99.9% certain pSAR_10g_ upper bound (dashed blue line); and the green line denotes the corrected pSAR_10g_ by the CSM. The LSF and the CSM are determined using the validation set (validation set: cyan dots; test set: black dots). E, Bar plot of the mean pSAR_10g_ overestimation error for each pSAR_10g_ correction method and each driving mode

**Figure 7:**
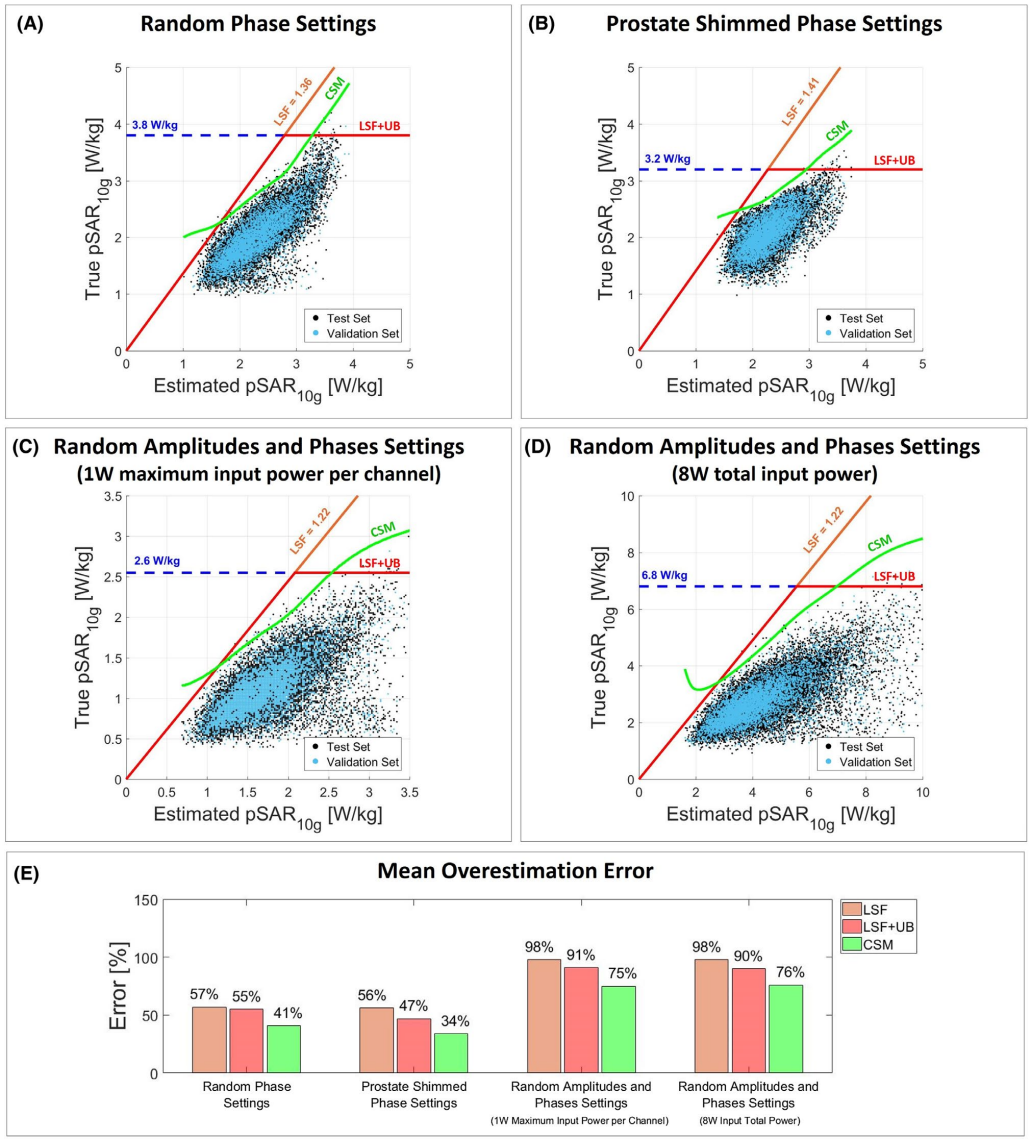
Deep learning. Scatter plot of true versus estimated pSAR_10g_ with random phases (A), prostate shimmed phase settings (B), random amplitudes and phases normalized to 1W maximum input power per channel (C), and random amplitudes and phases normalized to 8W input total power (D). The orange line denotes the corrected pSAR_10g_ by the LSF; the red line denotes the corrected pSAR_10g_ by the LSF limited by the 99.9% certain pSAR_10g_ upper bound (dashed blue line); and the green line denotes the corrected pSAR_10g_ by the CSM. The LSF and the CSM are determined using the validation set (validation set: cyan dots; test set: black dots). E, Bar plot of the mean pSAR_10g_ overestimation error for each pSAR_10g_ correction method and each driving mode

Figure 4 presents the scatter plot of true versus estimated pSAR_10g_ and the mean overestimation considering the model library pSAR_10g_ estimation method. Figures 5 and 6 present the same results for the model selection and multiple models selection methods, and Figure 7 presents the same plot for the deep learning method. The histogram of the pSAR_10g_ estimation error of each pSAR_10g_ estimation method, each driving mode, and each pSAR_10g_ correction method is presented in Supporting information Figures S9‐S13.

**Figure 8:**
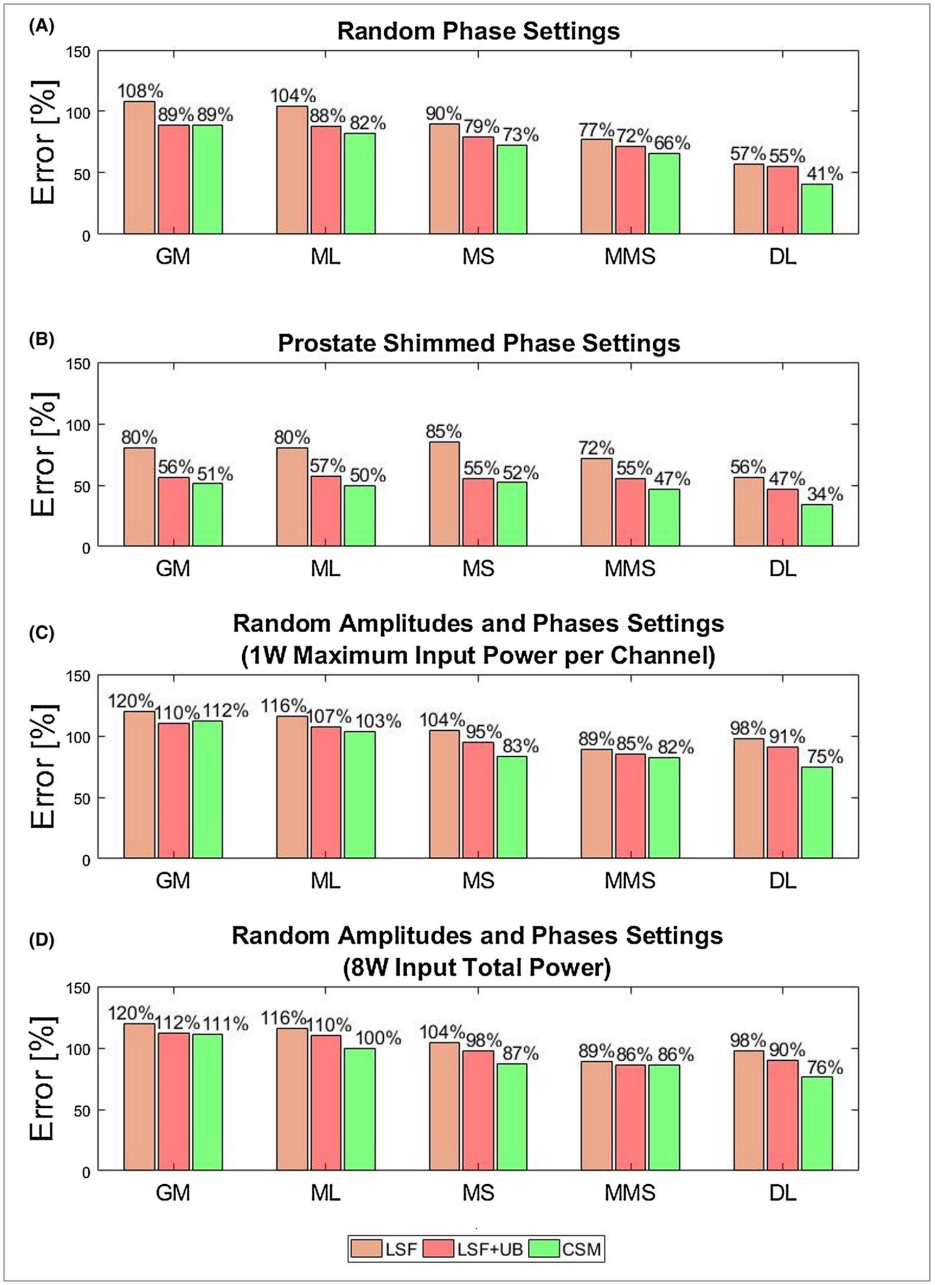
Bar plot of the mean overestimation error for each pSAR_10g_ estimation method with random phases (A), prostate shimmed phase settings (B), random amplitudes and phases normalized to 1W maximum input power per channel (C), and random amplitudes and phases normalized to 8W input total power (D). The orange bar denotes the mean overestimation of the corrected pSAR_10g_ by the LSF; the red bar denotes the mean overestimation of the corrected pSAR_10g_ by the LSF limited by the 99.9% certain pSAR_10g_ upper bound; and the green bar denotes the mean overestimation of the corrected pSAR_10g_ by the CSM. Abbreviations: DL, deep learning model; GM, general body model; ML, model library; MMS, multiple models selection; MS, model selection

The bar diagram in Figure 8 makes it easy to compare the pSAR_10g_ estimation methods covered by this study. It shows the mean overestimation after the correction for all considered pSAR_10g_ estimation methods for each driving mode. The deep learning–based pSAR_10g_ estimation method outperforms conventional methods. Compared with the pSAR_10g_ estimation methods based on the selection of the most similar models from a database, after the application of the CSM, it achieves a mean overestimation reduction of 44%‐28% for phase shimming and 13%‐9% for amplitude and phase shimming. The multiple models selection and the model selection methods are the second and third best pSAR_10g_ estimation methods, respectively.

## 5 DISCUSSION

The linear safety factor is a common approach to avoid potential underestimation due to errors in predicted peak local SAR levels.^10^, ^11^, ^12^, ^23^, ^24^ This work has shown that the linear safety factor results in unnecessarily severe scanning constraints. The correction of estimated peak local SAR levels (pSAR_10g_) using a linear safety factor produces an overconservative pSAR_10g_ prediction, even when the safety factor is defined with outlier rejection and a low underestimation probability is allowed.^27^ This is because for high estimated pSAR_10g_ values, the corrected pSAR_10g_ values are unreasonably high. If we consider the mean estimated pSAR_10g_ value with almost all methods, it already produces corrected pSAR_10g_ values higher than the 99.9% certain pSAR_10g_ upper bound.

Therefore, it could be reasonable to limit the corrected pSAR_10g_ values to this upper bound. This produces a slightly higher probability of underestimation (< 0.2%) but severely reduces the mean overestimation. Such a combination of corrected pSAR_10g_ values is in principle a first attempt at defining variable margin factors depending on the estimated SAR level.

However, a more appropriate way of tackling this problem is by making use of probability theory, to take into account the statistical distribution of realistically attainable pSAR_10g_ values. Therefore, a new approach to define a variable safety margin based on the conditional probability distribution was presented. This approach allows one to define a CSM to obtain, for each estimated pSAR_10g_ value, a corrected pSAR_10g_ value that will allow for only a very small predefined probability of underestimation (eg, 0.1%). The 0.1% probability pSAR_10g_ threshold violations will be modest with rapidly decreasing likelihood for higher pSAR_10g_ levels. Nevertheless, some users may prefer using the proposed approach with lower underestimation probabilities, such as 0.001% (Supporting information Figure S14).

This approach can be applied to any pSAR_10g_ estimation method. It was applied to five state‐of‐the‐art pSAR_10g_ estimation methods for a multitransmit array of eight fractionated dipole antennas for prostate imaging at 7 T^29^, ^30^ with three different drive modes: random phase settings, prostate shimmed phase settings, and random amplitude and phase settings. For each examined case, 23 × 250 validation sets were used to determine the linear safety factor and the CSM. A larger number of validation sets did not produce a significant change in the determined safety factor nor in performance (Supporting Information Figure S15).

Compared with the linear safety factor limited by the 99.9% certain pSAR_10g_ upper bound, the performance increase achievable with this method depends on the accuracy of the used pSAR_10g_ estimation method. With pSAR_10g_ estimation methods that show a good correlation between estimated and true pSAR_10g_ values (eg, deep learning method), the proposed approach allows a significant reduction of the mean pSAR_10g_ overestimation (16%‐28%). For methods that have a poor performance in predicting the true peak local SAR level (one generic model or worst‐case of all models), the benefit of the proposed approach is much less pronounced. For these methods, the reduction in mean pSAR_10g_ overestimation is less than 12%.

Note that a special case exists for extremely poor estimation methods. In that case, the estimated and true pSAR_10g_ values exhibit low correlation, or, in other words, are essentially independent; in this case, the joint probability is the product of their marginal probabilities *P* (*pSAR^E^*, *pSAR^T^*) = *P* (*pSAR^E^*)*P*(*pSAR^T^*). Then, the conditional probability coincides with the marginal probability of true value *P*(*pSAR^T^*|*pSAR^E^*) = *P*(*pSAR^E^*, *pSAR^T^*)/*P*(*pSAR^E^*) *=* *P*(*pSAR^T^*). This means that from the knowledge of the estimated value, nothing can be inferred, and if the CSM is defined considering ε=0.001, for any estimated pSAR_10g_ the CSM will determine a corrected pSAR_10g_ value that will approximately coincide with the 99.9% certain pSAR_10g_ upper bound. This is more or less the case for pSAR_10g_ prediction using one generic model with random phase settings. As indicated in Figure 3A, the scatter cloud shows very poor correlation of true pSAR_10g_ versus estimated pSAR_10g_. Therefore, the CSM results in an almost horizontal line following the 3.8 W/kg 99.9% confident upper limit.

Note that, for low estimated pSAR_10g_ values, the linear safety factor produces lower corrected pSAR_10g_ values than the conditional safety factor. Indeed, with the linear safety factor, the probability of underestimation is larger than 0.1% when a low pSAR_10g_ value is estimated, whereas with the CSM it is 0.1% for any estimated pSAR_10g_ value. Likewise, with the 99.9% certain pSAR_10g_ upper bound, the probability of underestimation is larger than 0.1% when a high pSAR_10g_ value is estimated. Moreover, because the histogram of estimated pSAR_10g_ values follows a gamma distribution, close to the range boundaries, the number of estimated pSAR_10g_ values is less and less. With a very small number of samples, the estimated conditional probability density function is larger (greater uncertainty) and less accurate (because it is defined as the ratio between very small numbers, the relative error is larger). These are probably the causes of the observed large overestimation for low estimated pSAR_10g_ values and the anomalies in the conditional probability density functions outside of the feasibly estimated pSAR_10g_ ranges (Supporting Information Figures S4‐S8). Note that the relatively large overestimation of these low expected pSAR_10g_ values is not at all problematic, because their occurrence is quite rare.

It is worth noting that, for the random amplitudes and phases settings, we assumed that each drive vector is equally likely to occur. This assumption is probably far from the truth for many RF pulse design strategies. Therefore, for the considered RF pulse type, the probability of occurrence of the drive vectors should be assessed and taken into account for the validation set generation (similarly to what has been done for prostate shimmed phase settings).

Furthermore, it must be considered that the time‐dependent drive vector of these custom RF pulses produces a time‐dependent SAR distribution with the peak value in a different location for each time step. The CSM, as well as the linear safety factor, is a global factor that does not take into account the spatial distribution of the SAR. Nevertheless, it can be used to define a correction factor (CSM/pSAR^E^) to apply to the whole SAR distribution (or virtual observation points) for each time step (in the same way that the linear safety factor is usually applied). Subsequently, the SAR distributions are integrated over time, and then the global peak SAR value can be assessed.

Results on phase‐amplitude shimming have been presented for two normalization methods: normalization on maximum input power per channel and normalization on total input power. Both normalizations resulted in different scatter clouds and different CSM curves. However, the performance in terms of mean overestimation was basically equivalent (Figure 8).

In addition, a comparison was made among the state‐of‐the‐art pSAR_10g_ estimation methods. We showed that when using a single generic body model for pSAR_10g_ estimation, a poor relation between estimated and true pSAR_10g_ is observed and the mean overestimation error after the correction with the CSM is 89% for random phase settings, 51% for prostate shimmed phase settings, 112% for random amplitude and phase settings normalized to 1W maximum input power per channel, and 111% for random amplitude and phase settings normalized to 8W input total power (Figure 8). Figure 3 clearly shows that the point cloud does not follow the diagonal and the corrected pSAR_10g_ values, almost coincident with the 99.9% certain pSAR_10g_ upper bound. Thus, the proposed correction approach allows a little mean overestimation reduction (less than 10%).

Similar results were found using a model library to predict pSAR_10g_ over all models. This pSAR_10g_ estimation is obviously more conservative and requires smaller linear safety factors (Figure 4). However, after the correction the mean overestimation was comparable with the generic body model method (82%, 50%, 103%, and 100%). In this case, the CSM allows a mean overestimation reduction from 4% to 12%, because if a low pSAR_10g_ value is estimated over all models, it is less likely that a high true pSAR_10g_ value is observed, and this weak relationship is exploited by the proposed correction method.

The pSAR_10g_ estimation methods based on the selection of the most similar model(s) from a database show better performance than the conventional estimation methods. If the most “similar” model is used, the pSAR_10g_ estimation is more accurate but not very precise (Figure 5). To manage the low precision due to the intersubject pSAR_10g_ variability, a considerable linear safety factor is required. Nevertheless, the model selection method achieves a lower mean overestimation after the correction with the proposed approach (73%, 52%, 83%, and 87%).

The use of multiple models reduces the probability of underestimation due to the intersubject pSAR_10g_ variability, and allows us to reduce the required safety factors. This results in an even lower mean overestimation error (66%, 47%, 82%, and 86%). With the CSM, an overestimation reduction up to 15% is reached with these estimation methods.

The proposed pSAR_10g_ correction method exploits the correlation between estimated and true pSAR_10g_ levels. Therefore, with more accurate and precise pSAR_10g_ estimation methods, the deep learning method provides the best performance (Figure 7). It reduces the mean pSAR_10g_ overestimation by almost 30%, achieving a mean overestimation error of 41% for random phase settings and 34% for prostate shimmed phase settings.

Considerable mean overestimation is still observed for random amplitude and phase shimming (75%‐76%). However, it should be noted that the neural network was trained with only phase‐shimmed training samples.^27^ This means that better performance might be obtained by training the network with amplitude and phase‐shimmed training samples. Nonetheless, even in this case the CSM allows one to reduce the mean overestimation of almost 20%.

It should also be noted that the deep learning–based method makes use of a network that was trained by penalizing the underestimation error more.^27^ This was done in an attempt to avoid underestimation, yet some underestimation error remained. Therefore, the method was included in this study as one of the investigated SAR assessment methods. If the network is trained by equally penalizing the underestimation and overestimation error, the deep learning–based method would have shown a larger degree of underestimation. The points in the scatter clouds in Figure 7 would shift to the left, the linear safety factor required to avoid underestimation would increase (red lines in Figure 7 become steeper), and the linear safety factor approach would result in a larger overestimation. Therefore, the benefit of the CSM approach would appear larger. We chose to use the deep learning–based method as it was published, which results in a conservative estimate of what benefit the CSM could provide for such methods.

## 6 CONCLUSIONS

To avoid underestimation of predicted peak local SAR (pSAR_10g_) levels for multitransmit arrays, a safety factor is often applied to correct (increase) the predicted pSAR_10g_ levels. This work has shown that this approach results in drastic and unnecessary overestimation of pSAR_10g_ levels, particularly if the estimated pSAR_10g_ level is relatively high.

In this work, an alternative approach for safety margin definition is presented using probability theory. This CSM approach allows us to define a variable safety margin for each possible estimated pSAR_10g_ value.

For prostate imaging at 7 T, the proposed CSM approach results in lower mean overestimation for all investigated local SAR estimation methods. Compared with the linear safety factor in combination with the 99.9% upper bound, the reduction of overestimation up to 30% is reached for the more accurate local SAR assessment methods.

## CONFLICT OF INTEREST

Mr. Meliadò is an employee of Tesla Dynamic Coils.

## Supporting information


**FIGURE S1** Transverse maximum intensity projection of the worst‐case peak 10*g* average specific absorption rate (pSAR_10g_) distributions with uniform amplitude (8 × 1W input power)
**FIGURE S2** Root‐mean‐square error (RMSE) matrix. Each entry RMSE [*n,m*] represents the RMSE between the worst‐case pSAR_10g_ distribution of the model *n* and the registered worst‐case pSAR_10g_ distribution of the model *m*
**FIGURE S3** Worst‐case pSAR_10g_ distribution of each model and registered worst‐case pSAR_10g_ distribution of the most representative “local SAR model”
**FIGURE S4** Generic body model (validation set): scatter plot of the true pSAR^T^ versus the estimated pSAR^E^ (first column); marginal probability density function *f_E_(pSAR^E^)* of the estimated pSAR values (second column); joint probability density function *f_E,T_(pSAR^E^,* *pSAR^T^)* of the estimated and true pSAR values (third column); and 2D conditional probability density function *f_T|E_(pSAR^T^|pSAR^E^)* obtained by combining the conditional probability density functions for each possible pSAR^E^ value (fourth column)
**FIGURE S5** Model library (validation set): scatter plot of the true pSAR^T^ versus the estimated pSAR^E^ (first column); marginal probability density function *f_E_(pSAR^E^)* of the estimated pSAR values (second column); joint probability density function *f_E,T_(pSAR^E^,* *pSAR^T^)* of the estimated and true pSAR values (third column); and 2D conditional probability density function *f_T|E_(pSAR^T^|pSAR^E^)* obtained by combining the conditional probability density functions for each possible pSAR^E^ value (fourth column)
**FIGURE S6** Model selection (validation set): scatter plot of the true pSAR^T^ versus the estimated pSAR^E^ (first column); marginal probability density function *f_E_(pSAR^E^)* of the estimated pSAR values (second column); joint probability density function *f_E,T_(pSAR^E^,* *pSAR^T^)* of the estimated and true pSAR values (third column); and 2D conditional probability density function *f_T|E_(pSAR^T^|pSAR^E^)* obtained by combining the conditional probability density functions for each possible pSAR^E^ value (fourth column)
**FIGURE S7** Multiple models selection (validation set): scatter plot of the true pSAR^T^ versus the estimated pSAR^E^ (first column); marginal probability density function *f_E_(pSAR^E^)* of the estimated pSAR values (second column); joint probability density function *f_E,T_(pSAR^E^,* *pSAR^T^)* of the estimated and true pSAR values (third column); and 2D conditional probability density function *f_T|E_(pSAR^T^|pSAR^E^)* obtained by combining the conditional probability density functions for each possible pSAR^E^ value (fourth column)
**FIGURE S8** Deep learning (validation set): scatter plot of the true pSAR^T^ versus the estimated pSAR^E^ (first column); marginal probability density *f_E_(pSAR^E^)* of the estimated pSAR values (second column); joint probability density function *f_E,T_(pSAR^E^*, *pSAR^T^)* of the estimated and true pSAR values (third column); and 2D conditional probability density function *f_T|E_(pSAR^T^|pSAR^E^)* obtained by combining the conditional probability density functions for each possible pSAR^E^ value (fourth column)
**FIGURE S9** Generic body model (test set). Scatter plot of true versus estimated pSAR_10g_ and histogram of the pSAR_10g_ estimation error for each driving mode and each pSAR_10g_ correction method. The linear safety factor (LSF) and the conditional safety margin (CSM) were determined using the validation set (cyan dots)
**FIGURE S10** Model library (test set). Scatter plot of true versus estimated pSAR_10g_ and histogram of the pSAR_10g_ estimation error for each driving mode and each pSAR_10g_ correction method. The LSF and the CSM were determined using the validation set (cyan dots)
**FIGURE S11** Model selection (test set). Scatter plot of true versus estimated pSAR_10g_ and histogram of the pSAR_10g_ estimation error for each driving mode and each pSAR_10g_ correction method. The LSF and the CSM were determined using the validation set (cyan dots)
**FIGURE S12** Multiple models selection (test set). Scatter plot of true versus estimated pSAR_10g_ and histogram of the pSAR_10g_ estimation error for each driving mode and each pSAR_10g_ correction method. The LSF and the CSM were determined using the validation set (cyan dots)
**FIGURE S13** Deep learning (test set). Scatter plot of true versus estimated pSAR_10g_ and histogram of the pSAR_10g_ estimation error for each driving mode and each pSAR_10g_ correction method. The LSF and the CSM were determined using the validation set (cyan dots)
**FIGURE S14** A, Scatter plot of true versus estimated pSAR_10g_ using the deep learning method with random phase settings (test set: black dots; validation set: cyan dots). The orange line denotes the corrected pSAR_10g_ by the LSF based on the worst‐case ratio of true and estimated pSAR_10g_. The green line denotes the corrected pSAR_10g_ by the CSM with probability of underestimation of 0.001% (*ε* = 0.00001). B, Histogram of the pSAR_10g_ estimation error for the corrected pSAR_10g_ by the LSF. C, Histogram of the pSAR_10g_ estimation error for the corrected pSAR_10g_ by the CSM. The LSF and the CSM were determined using the validation set
**FIGURE S15** A, Scatter plot of true versus estimated pSAR_10g_ using the deep learning method with random phase settings (23 × 1000 validation sets: black dots; 23 × 250 validation sets: cyan dots). The orange line denotes the corrected pSAR_10g_ by the LSF determined with 23 × 1000 validation sets (LSF = 1.37). It practically coincides with the corrected pSAR_10g_ by the LSF determined with 23 × 250 validation sets (LSF = 1.36). The solid and dotted green lines denote the corrected pSAR_10g_ by the CSM determined with 23 × 1000 validation sets and with 23 × 250 validation sets, respectively (*ε* = 0.001). B, Histogram of the pSAR_10g_ estimation error for the corrected pSAR_10g_ by the LSF determined with 23 × 1000 validation sets. C, Histogram of the pSAR_10g_ estimation error for the corrected pSAR_10g_ by the CSM determined with 23 × 1000 validation sets
**TABLE S1** The five most representative local SAR models for each model
