# Supplementary material for: Conditional safety margins for less conservative peak local SAR assessment: A probabilistic approach
Source: Magn Reson Med. 2020 Jun 3;84(6):3379–95. doi: 10.1002/mrm.28335 (PMC7540599; doi:10.1002/mrm.28335)
Supplement: Supplementary file 1 — FIGURE S1 Transverse maximum intensity projection of the worst‐case peak 10g average specific absorption rate (pSAR10g) distributions with uniform amplitude (8 × 1W input power) FIGURE S2 Root‐mean‐square error (RMSE) matrix. Each entry RMSE [n,m] represents the RMSE between the worst‐case pSAR10g distribution of the model n and the registered worst‐case pSAR10g distribution of the model m FIGURE S3 Worst‐case pSAR10g distribution of each model and registered worst‐case pSAR10g distribution of the most representative “local SAR model” FIGURE S4 Generic body model (validation set): scatter plot of the true pSART versus the estimated pSARE (first column); marginal probability density function fE(pSARE) of the estimated pSAR values (second column); joint probability density function fE,T(pSARE, pSART) of the estimated and true pSAR values (third column); and 2D conditional probability density function fT|E(pSART|pSARE) obtained by combining the conditional probability density functions for each possible pSARE value (fourth column) FIGURE S5 Model library (validation set): scatter plot of the true pSART versus the estimated pSARE (first column); marginal probability density function fE(pSARE) of the estimated pSAR values (second column); joint probability density function fE,T(pSARE, pSART) of the estimated and true pSAR values (third column); and 2D conditional probability density function fT|E(pSART|pSARE) obtained by combining the conditional probability density functions for each possible pSARE value (fourth column) FIGURE S6 Model selection (validation set): scatter plot of the true pSART versus the estimated pSARE (first column); marginal probability density function fE(pSARE) of the estimated pSAR values (second column); joint probability density function fE,T(pSARE, pSART) of the estimated and true pSAR values (third column); and 2D conditional probability density function fT|E(pSART|pSARE) obtained by combining the conditional probability density functions for [file MRM-84-3379-s001.docx]

**Supporting Information:**

**Conditional safety margins for less conservative peak local SAR assessment: a probabilistic approach**

E.F. Meliadò, A. Sbrizzi, C.A.T van den Berg, B.R. Steensma, P.R. Luijten, and A.J.E Raaijmakers

Worst-Case Local SAR Distributions and RMSE Matrix:


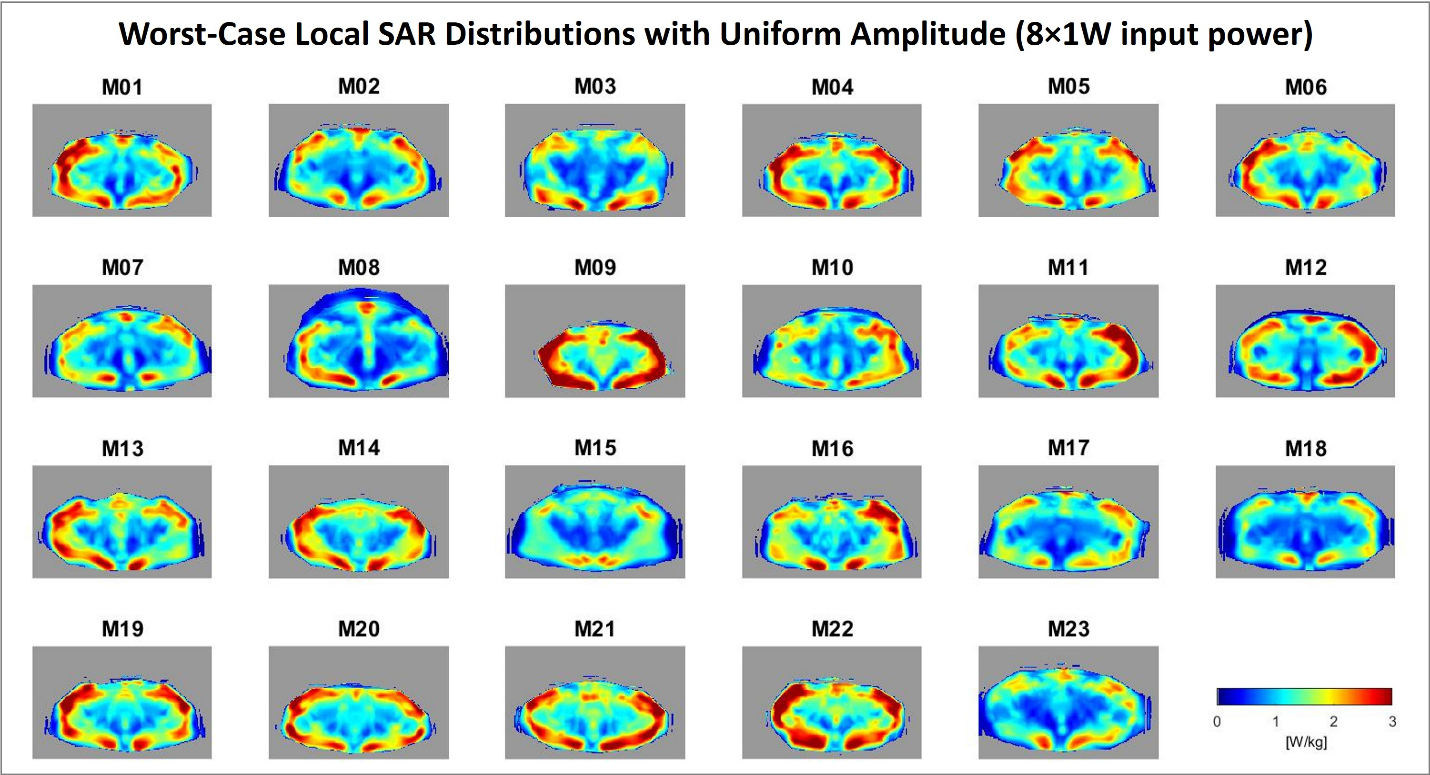


**Supporting Information Figure S1:** Transverse MIP of the worst-case SAR_10g_ distributions with uniform amplitude (8×1W input power).


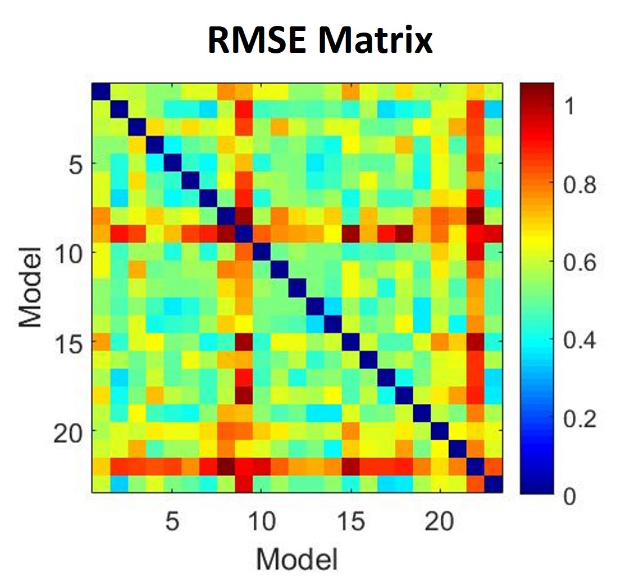


**Supporting Information Figure S2:** Root-mean-square error (RMSE) matrix. Each entry RMSE[*n,m*] represents the RMSE between the worst-case SAR_10g_ distribution of the model *n* and the registered worst-case SAR_10g_ distribution of the model *m.*

Model Selection: The Most Representative Local SAR Model


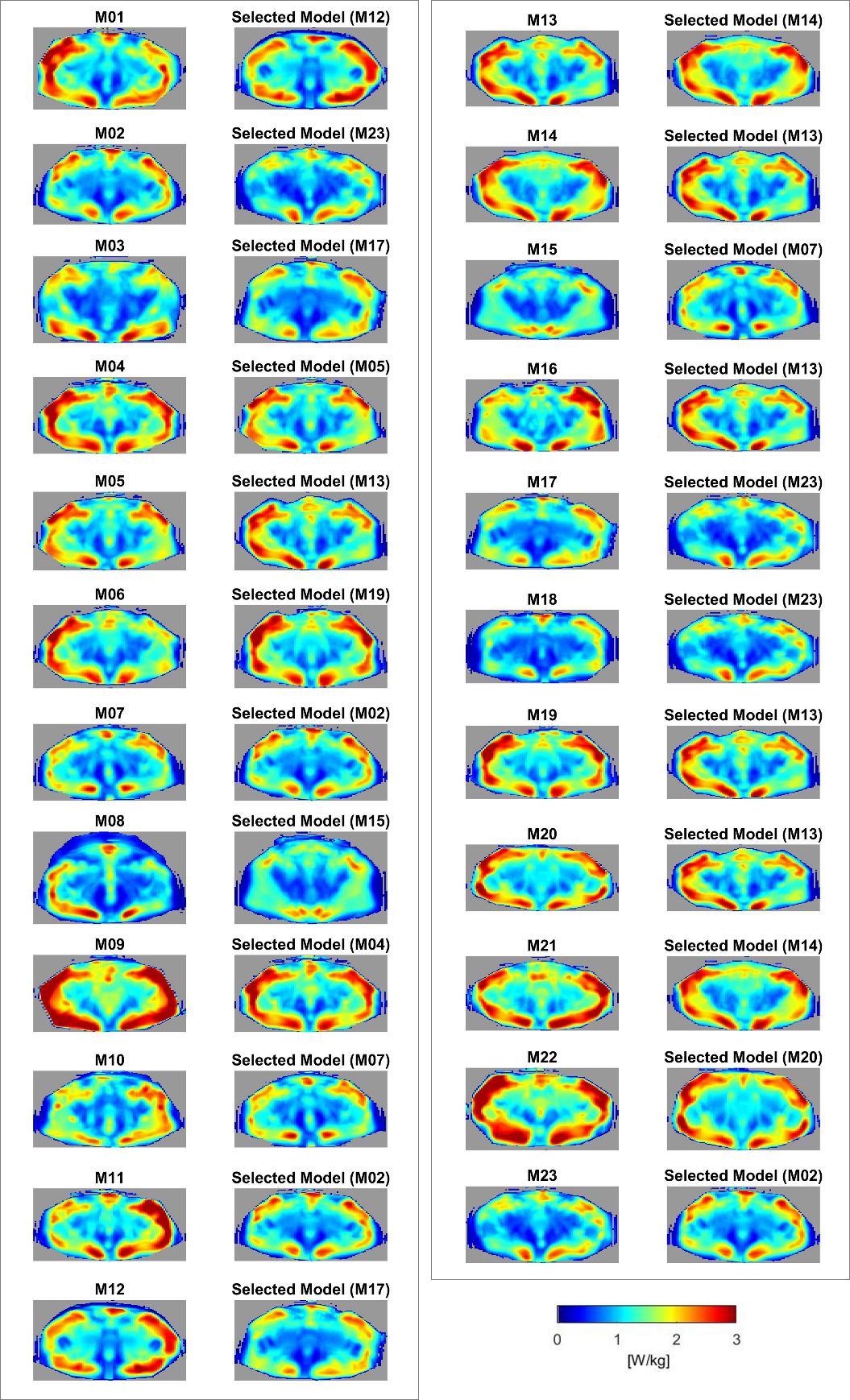


**Supporting Information Figure S3:** Worst-case SAR_10g_ distribution of each model and registered worst-case SAR_10g_ distribution of the most representative “local SAR model”*.*

Multiple Models Selection: The Five Most Representative Local SAR Models

**
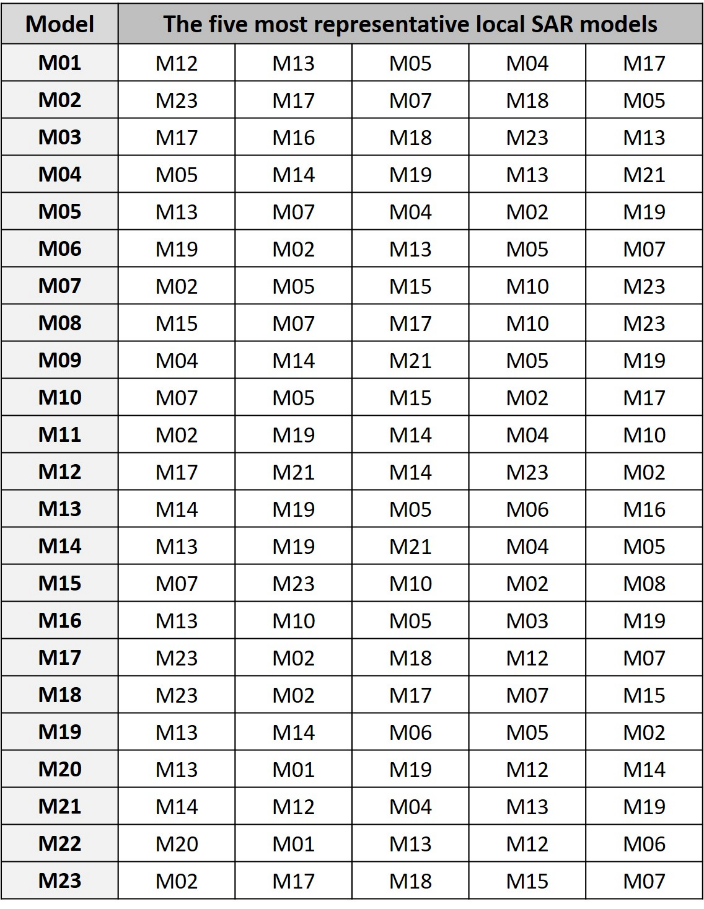
**

**Supporting Information Table S1:** The five most representative “local SAR models” for each model.

Probability Density Functions: Generic Body Model

**
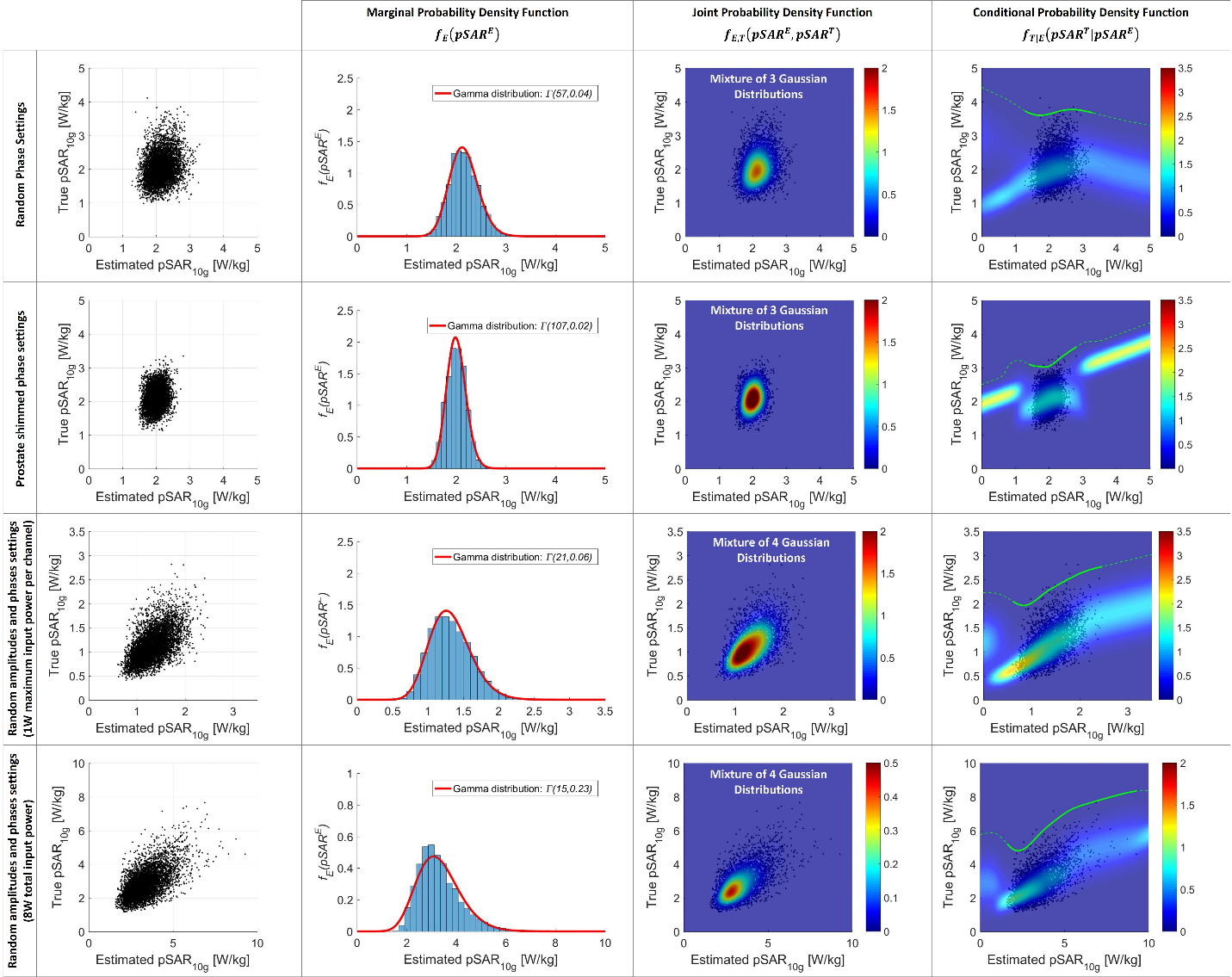
**

**Supporting Information Figure S4:** Generic Body Model (Validation Set) - Scatter plot of the true pSAR^T^ versus the estimated pSAR^E^ (first column). Marginal probability density function *f_E_(pSAR^E^)* of the estimated pSAR values (second column). Joint probability density function *f_E,T_(pSAR^E^, pSAR^T^)* of the estimated and true pSAR values (third column). 2D conditional probability density function *f_T|E_(pSAR^T^|pSAR^E^)* obtained combining the conditional probability density functions for each possible pSAR^E^ value (fourth column).

Probability Density Functions: Model Library


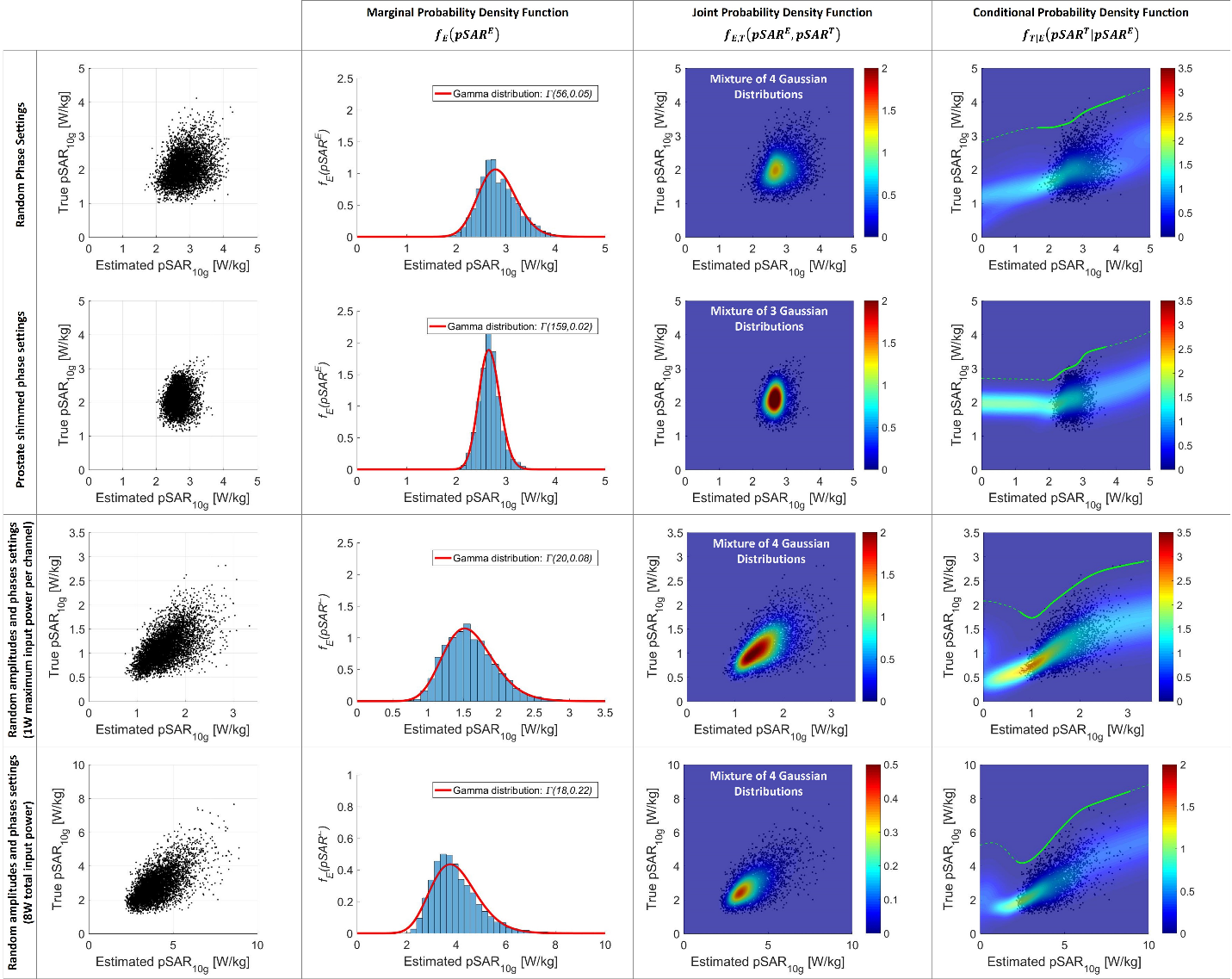


**Supporting Information Figure S5:** Model Library (Validation Set) - Scatter plot of the true pSAR^T^ versus the estimated pSAR^E^ (first column). Marginal probability density function *f_E_(pSAR^E^)* of the estimated pSAR values (second column). Joint probability density function *f_E,T_(pSAR^E^, pSAR^T^)* of the estimated and true pSAR values (third column). 2D conditional probability density function *f_T|E_(pSAR^T^|pSAR^E^)* obtained combining the conditional probability density functions for each possible pSAR^E^ value (fourth column).

Probability Density Functions: Model Selection


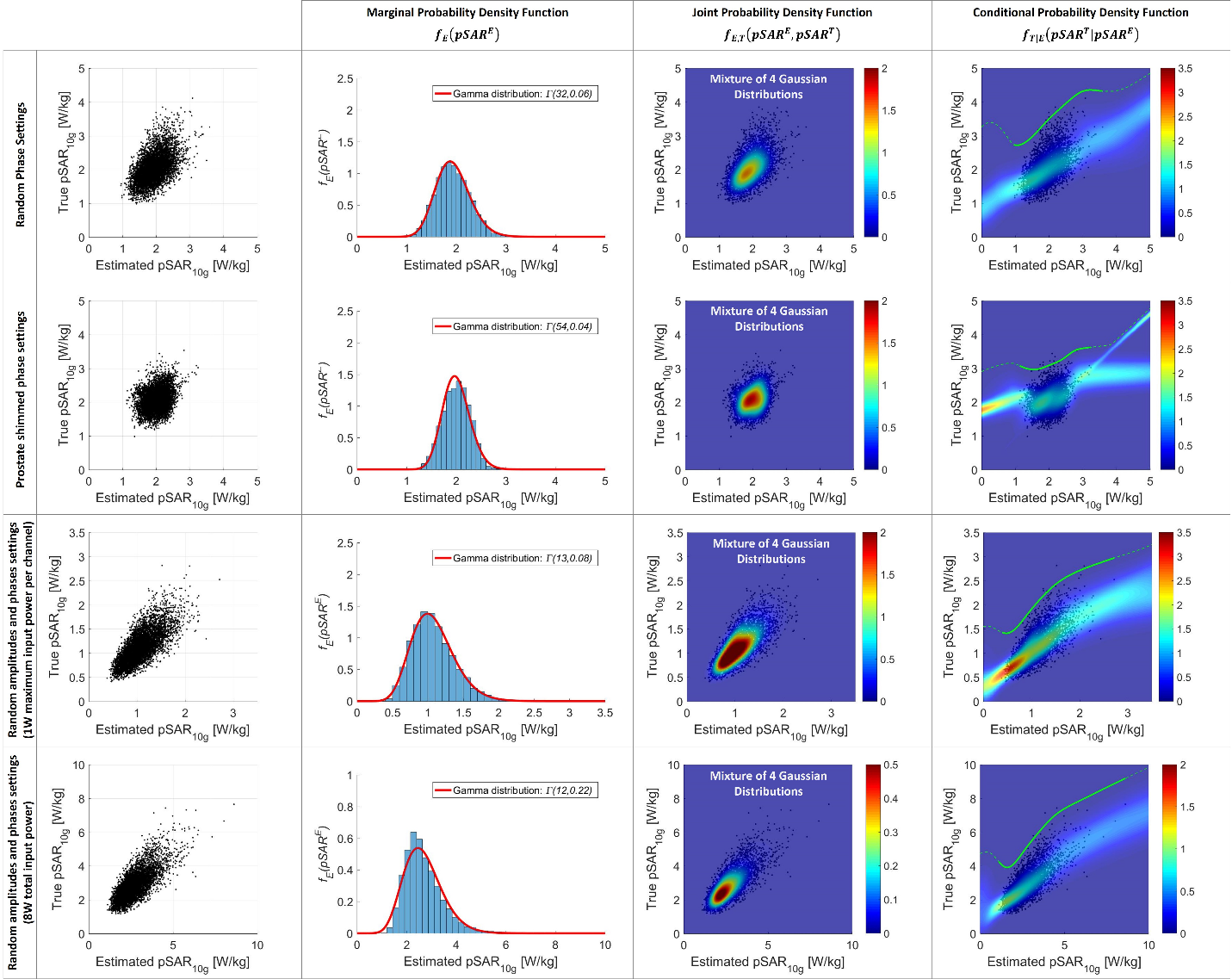


**Supporting Information Figure S6:** Model Selection (Validation Set) - Scatter plot of the true pSAR^T^ versus the estimated pSAR^E^ (first column). Marginal probability density function *f_E_(pSAR^E^)* of the estimated pSAR values (second column). Joint probability density function *f_E,T_(pSAR^E^, pSAR^T^)* of the estimated and true pSAR values (third column). 2D conditional probability density function *f_T|E_(pSAR^T^|pSAR^E^)* obtained combining the conditional probability density functions for each possible pSAR^E^ value (fourth column).

Probability Density Functions: Multiple Models Selection


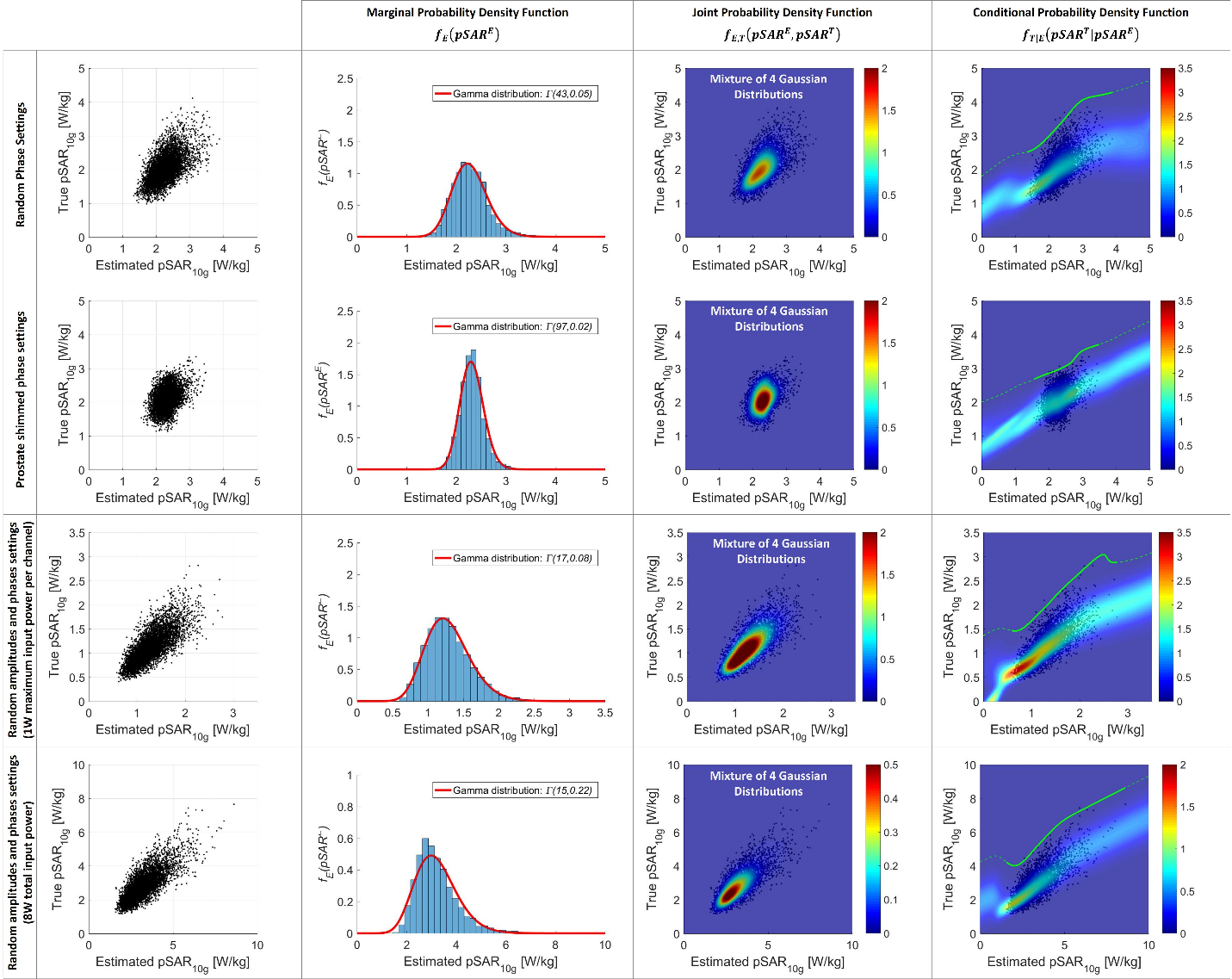


**Supporting Information Figure S7:** Multiple Models Selection (Validation Set) - Scatter plot of the true pSAR^T^ versus the estimated pSAR^E^ (first column). Marginal probability density function *f_E_(pSAR^E^)* of the estimated pSAR values (second column). Joint probability density function *f_E,T_(pSAR^E^, pSAR^T^)* of the estimated and true pSAR values (third column). 2D conditional probability density function *f_T|E_(pSAR^T^|pSAR^E^)* obtained combining the conditional probability density functions for each possible pSAR^E^ value (fourth column).

Probability Density Functions: Deep Learning


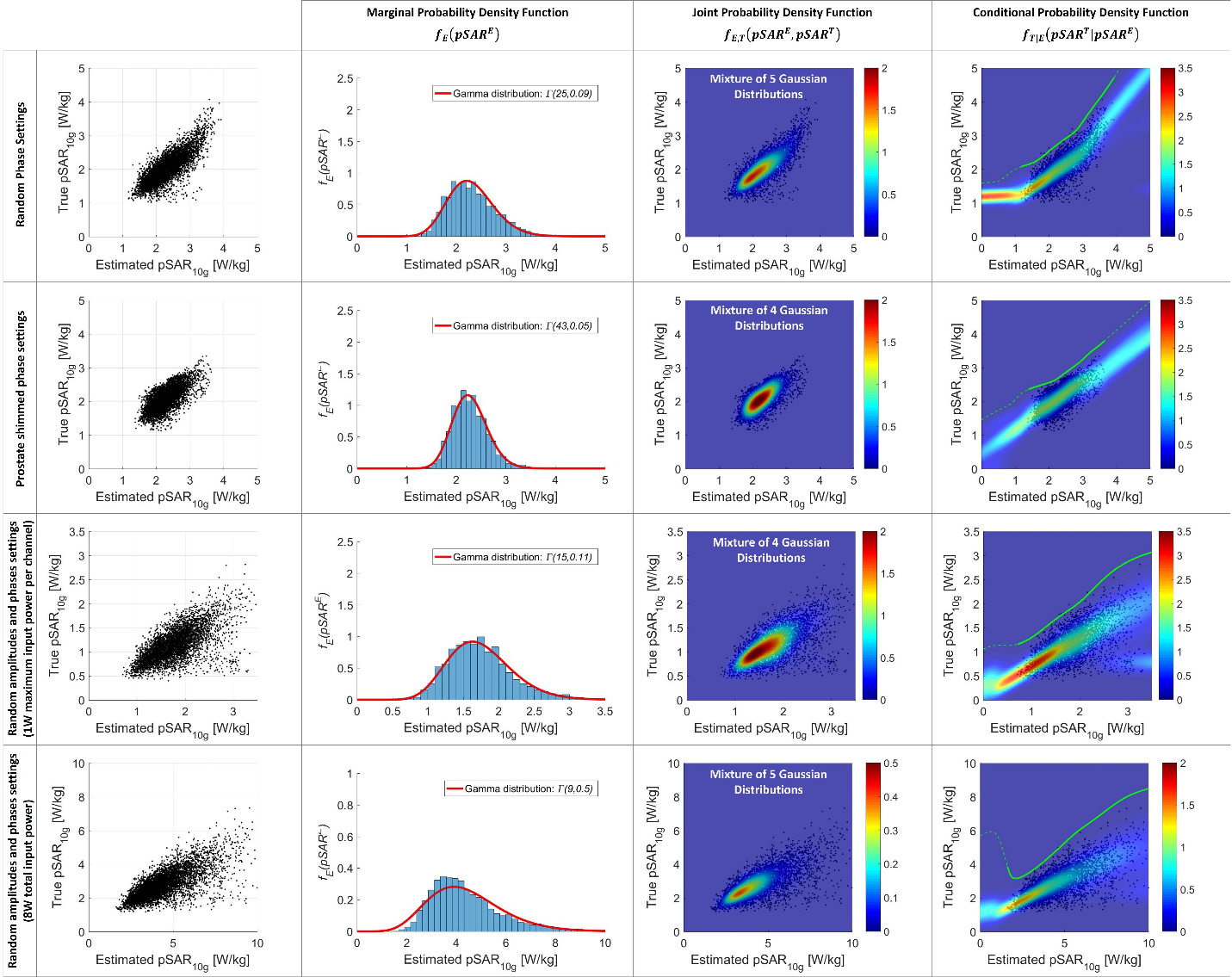


**Supporting Information Figure S8:** Deep Learning (Validation Set) - Scatter plot of the true pSAR^T^ versus the estimated pSAR^E^ (first column). Marginal probability density function *f_E_(pSAR^E^)* of the estimated pSAR values (second column). Joint probability density function *f_E,T_(pSAR^E^, pSAR^T^)* of the estimated and true pSAR values (third column). 2D conditional probability density function *f_T|E_(pSAR^T^|pSAR^E^)* obtained combining the conditional probability density functions for each possible pSAR^E^ value (fourth column).

Histogram of the pSAR_10g_ Estimation Error: Generic Body Model


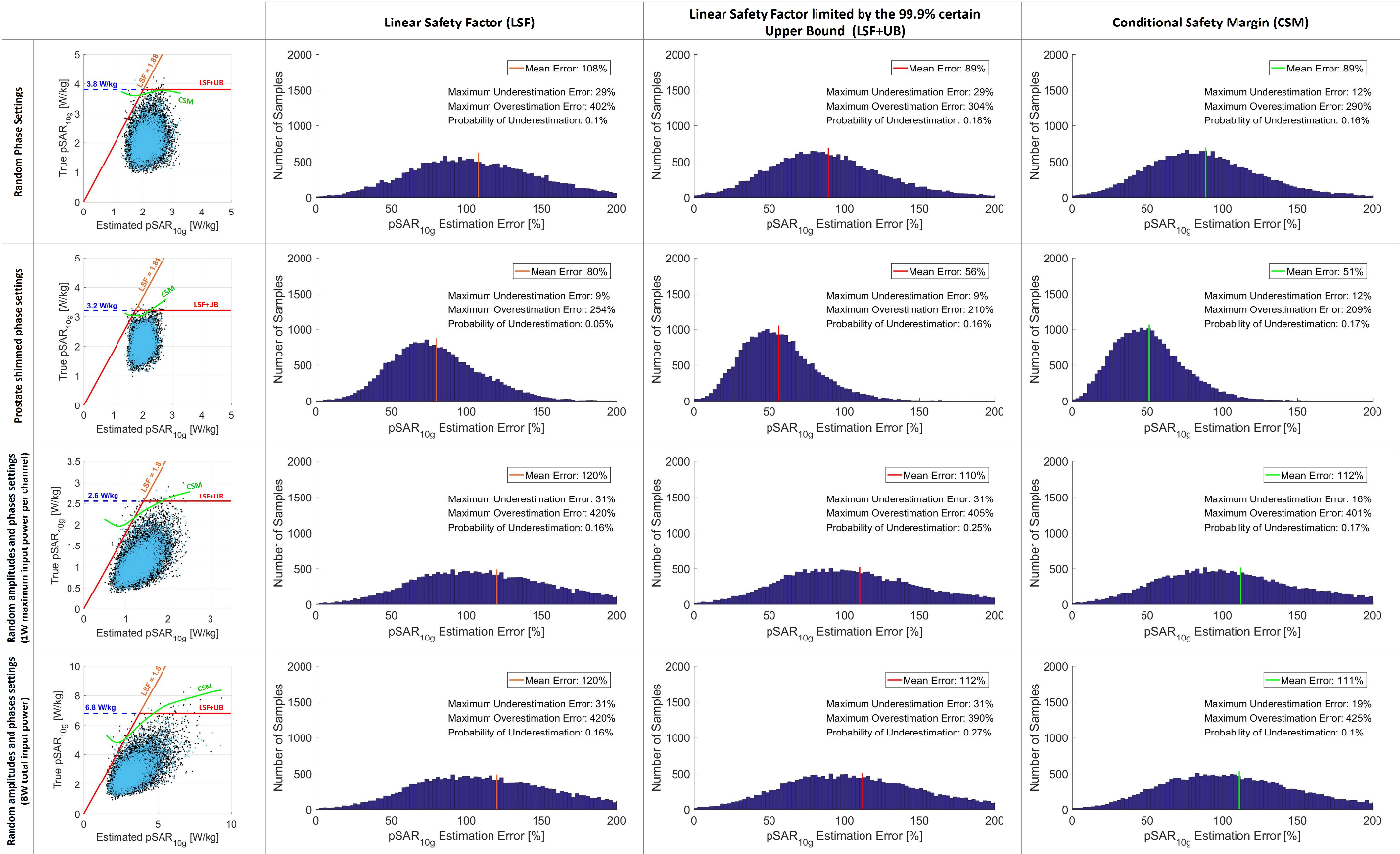


**Supporting Information Figure S9:** Generic Body Model (Test Set) - Scatter plot of true versus estimated pSAR_10g_ and histogram of the pSAR_10g_ estimation error for each driving mode and each pSAR_10g_ correction method. The linear safety factor and the conditional safety margin are determined using the validation set (cyan dots).

Histogram of the pSAR_10g_ Estimation Error: Model Library


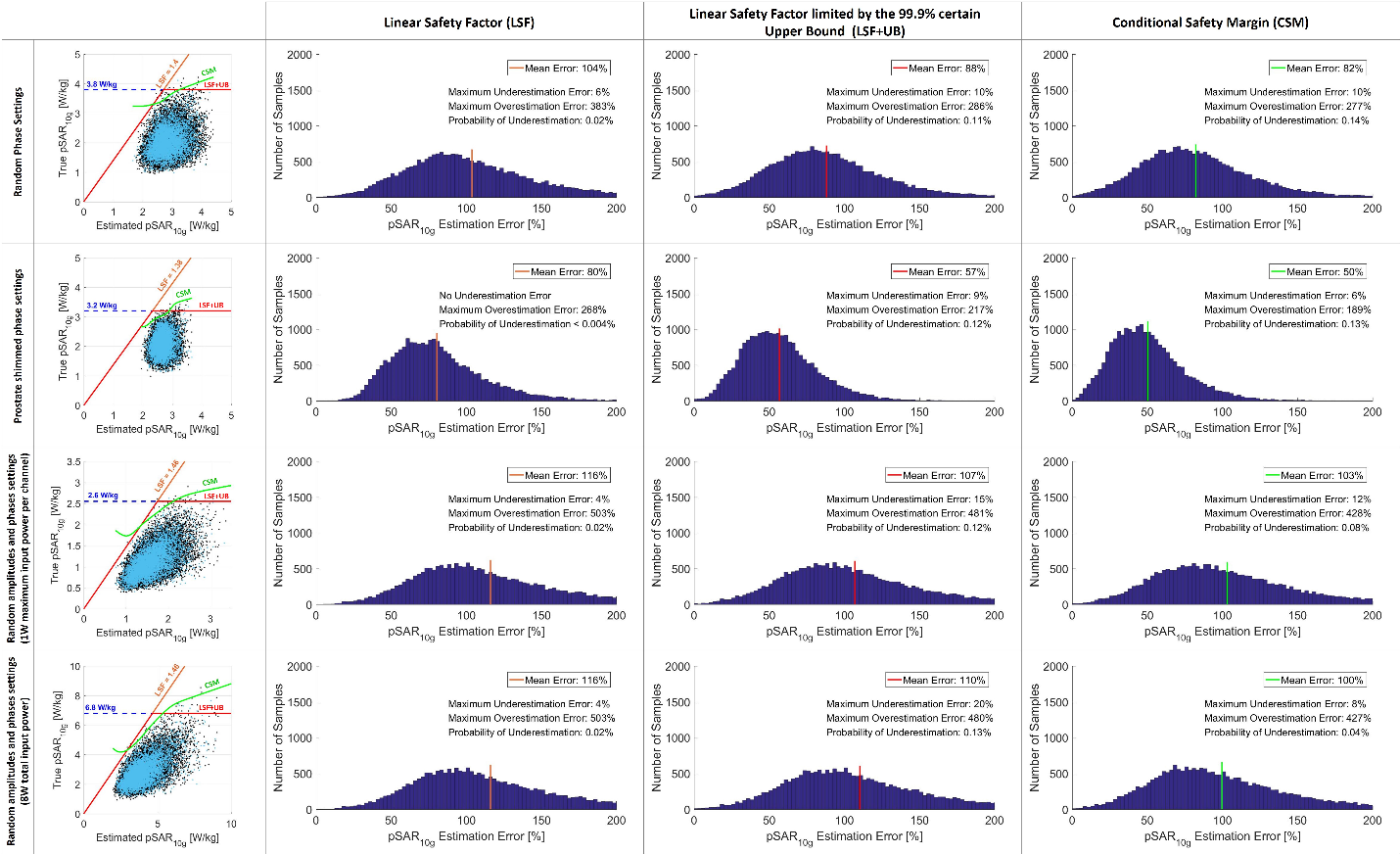


**Supporting Information Figure S10:** Model Library (Test Set) - Scatter plot of true versus estimated pSAR_10g_ and histogram of the pSAR_10g_ estimation error for each driving mode and each pSAR_10g_ correction method. The linear safety factor and the conditional safety margin are determined using the validation set (cyan dots).

Histogram of the pSAR_10g_ Estimation Error: Model Selection


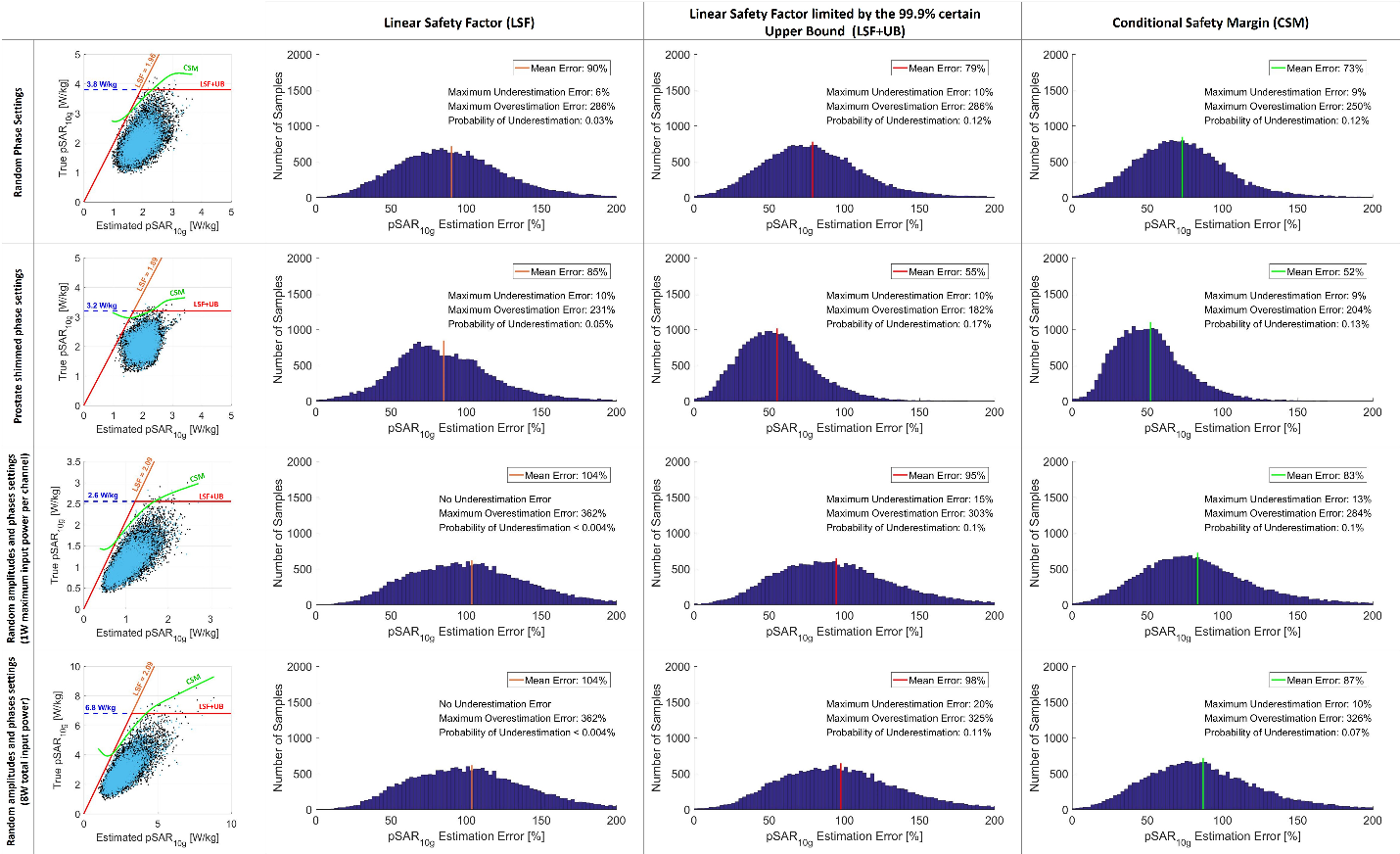


**Supporting Information Figure S11:** Model Selection (Test Set) - Scatter plot of true versus estimated pSAR_10g_ and histogram of the pSAR_10g_ estimation error for each driving mode and each pSAR_10g_ correction method. The linear safety factor and the conditional safety margin are determined using the validation set (cyan dots).

Histogram of the pSAR_10g_ Estimation Error: Multiple Models Selection


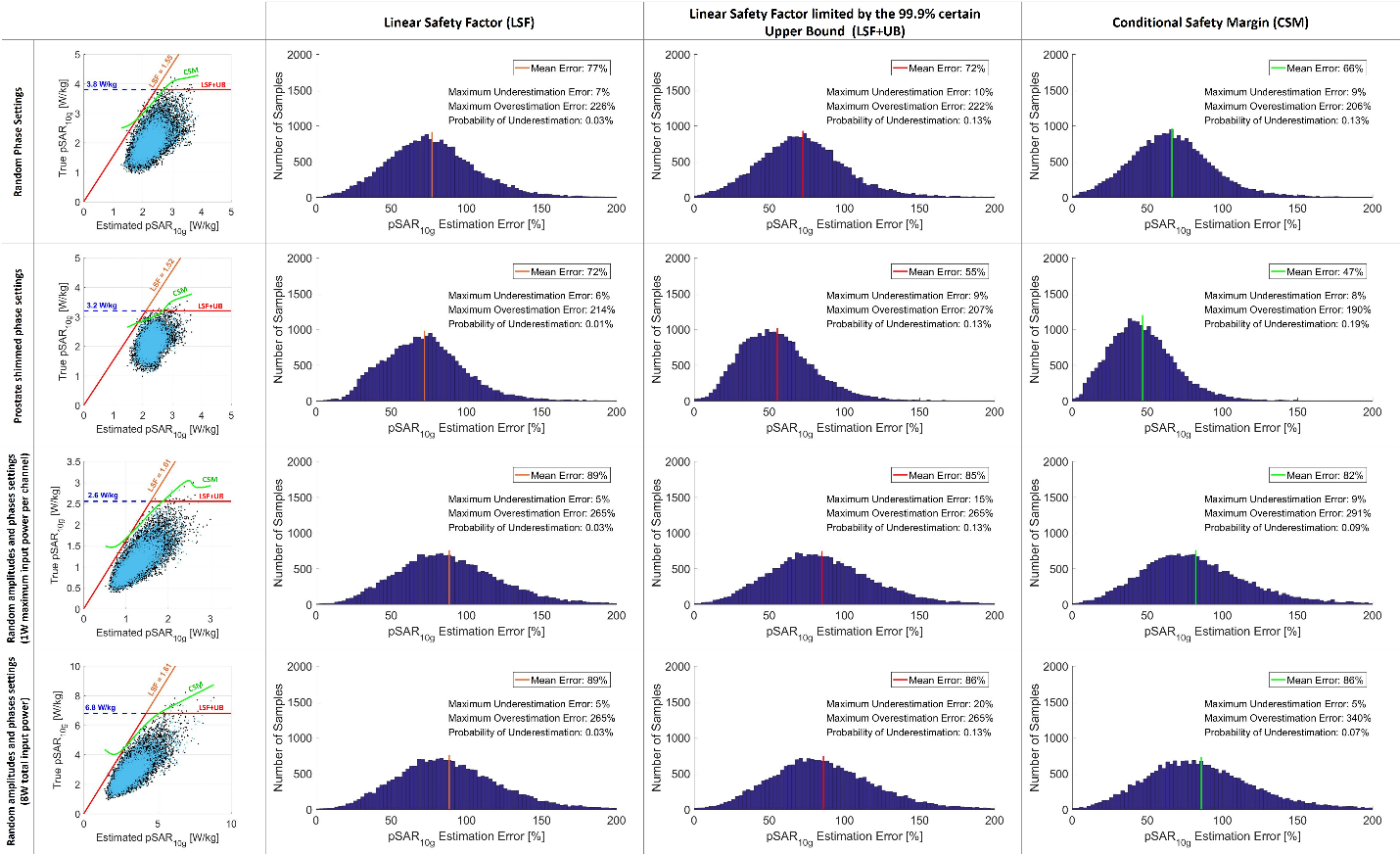


**Supporting Information Figure S12:** Multiple Models Selection (Test Set) - Scatter plot of true versus estimated pSAR_10g_ and histogram of the pSAR_10g_ estimation error for each driving mode and each pSAR_10g_ correction method. The linear safety factor and the conditional safety margin are determined using the validation set (cyan dots).

Histogram of the pSAR_10g_ Estimation Error: Deep Learning


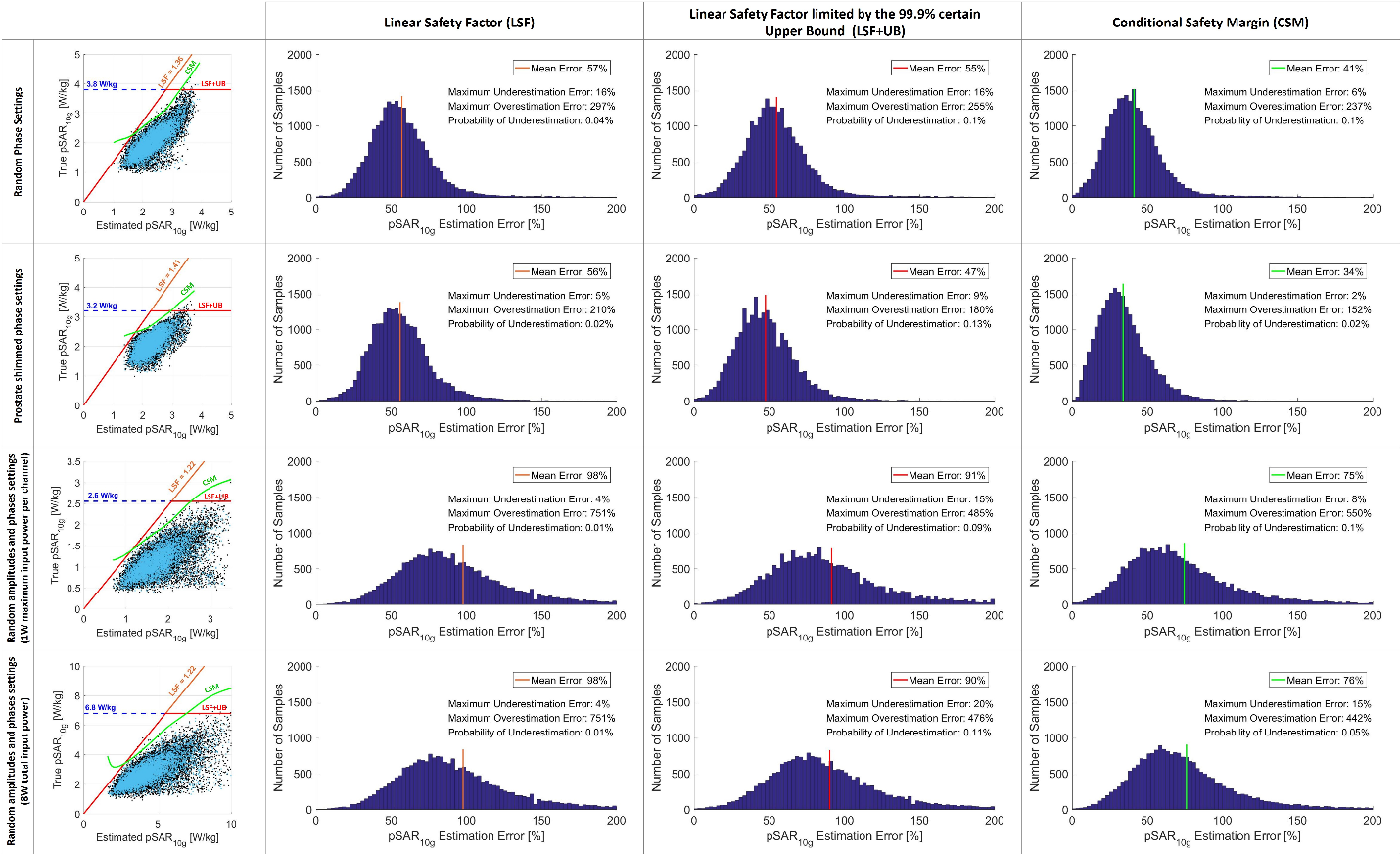


**Supporting Information Figure S13:** Deep Learning (Test Set) - Scatter plot of true versus estimated pSAR_10g_ and histogram of the pSAR_10g_ estimation error for each driving mode and each pSAR_10g_ correction method. The linear safety factor and the conditional safety margin are determined using the validation set (cyan dots).

Conditional Safety Margin with Very Low Probability of Underestimation (P=0.001%):


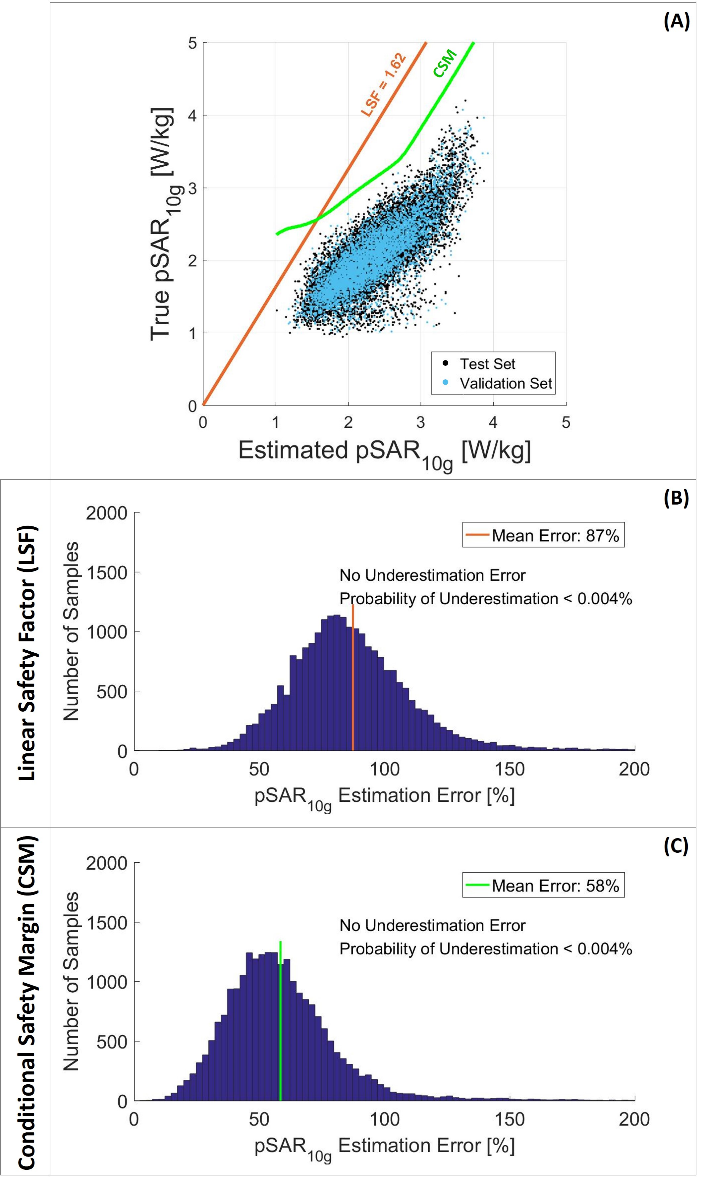


**Supporting Information Figure S14:** (A) Scatter plot of true versus estimated pSAR_10g_ using the deep learning method with random phase settings (test set: black dots – validation set: cyan dots). The orange line is the corrected pSAR_10g_ by the linear safety factor based on the worst case ratio of true and estimated pSAR_10g_. The green line is the corrected pSAR_10g_ by the conditional safety margin with probability of underestimation of 0.001% (ε = 0.00001). (B) The histogram of the pSAR_10g_ estimation error for the corrected pSAR_10g_ by the linear safety factor. (C) The histogram of the pSAR_10g_ estimation error for the corrected pSAR_10g_ by the conditional safety margin.

The linear safety factor and the conditional safety margin are determined using the validation set.

Linear Safety Factor and Conditional Safety Margin determined with 23×1000 Validation Sets:


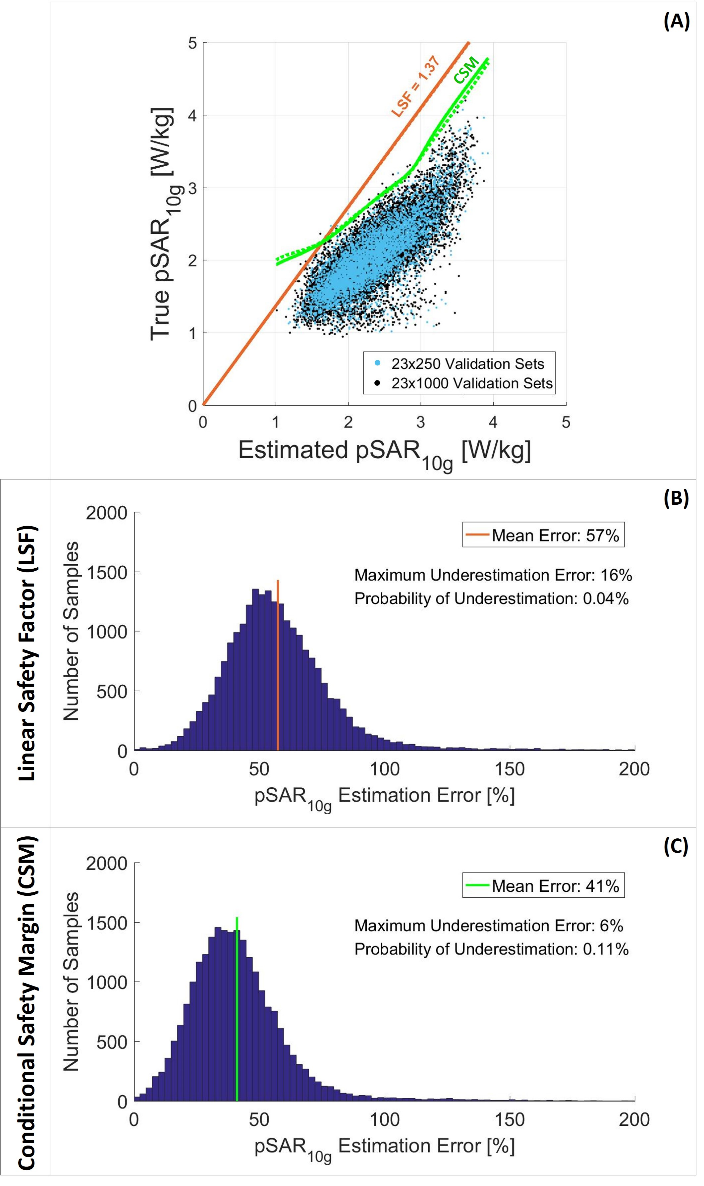


**Supporting Information Figure S15:** (A) Scatter plot of true versus estimated pSAR_10g_ using the deep learning method with random phase settings (23×1000 validation sets: black dots – 23×250 validation sets: cyan dots). The orange line is the corrected pSAR_10g_ by the linear safety factor determined with 23×1000 validation sets (LSF = 1.37). It practically coincides with the corrected pSAR_10g_ by the linear safety factor determined with 23×250 validation sets (LSF = 1.36). The solid and dotted green lines are the corrected pSAR_10g_ by the conditional safety margin determined with 23×1000 validation sets and with 23×250 validation sets respectively (ε = 0.001). (B) The histogram of the pSAR_10g_ estimation error for the corrected pSAR_10g_ by the linear safety factor determined with 23×1000 validation sets. (C) The histogram of the pSAR_10g_ estimation error for the corrected pSAR_10g_ by the conditional safety margin determined with 23×1000 validation sets.
